# The Role of Collagen Genetic Variability in Degenerative Disc Disease and Related Conditions

**DOI:** 10.2174/0113892029385458260414060252

**Published:** 2026-04-17

**Authors:** Adulrahman M. Bani Khaled, Abdelrahim Alqudah, Esam Qnais, Badriyah S Alotaibi, Baker Al-Shara, Alaa AA Aljabali

**Affiliations:** 1 Department of Biology and Biotechnology, Faculty of Science, The Hashemite University, Zarqa, Jordan;; 2 Department of Clinical Pharmacy and Pharmacy Practice, Faculty of Pharmaceutical Sciences, The Hashemite University, Zarqa, Jordan;; 3 Department of Pharmaceutical Sciences, College of Pharmacy, Princess Nourah bint Abdulrahman University, P.O. Box 84428, Riyadh, 11671, Saudi Arabia;; 4 Department of Medical Laboratory Sciences, Faculty of Allied Medical Sciences, Jadara University, Irbid, Jordan;; 5 Department of Pharmaceutics and Pharmaceutical Technology, Faculty of Pharmacy, Yarmouk University, Irbid, 21163, Jordan

**Keywords:** Degenerative Disc Disease (DDD), CRISPR/Cas9 therapeutics, collagen genetic polymorphisms, intervertebral disc

## Abstract

Degenerative Disc Disease (DDD) and related musculoskeletal disorders depend heavily on the structural integrity of collagen in the Extracellular Matrix (ECM) of connective tissues. The roles of collagen types I, II, IX, and XI in Intervertebral Disc (IVD) dynamics have been previously examined. Genetic polymorphisms in collagen-encoding genes are associated with susceptibility to DDD. These polymorphisms influence the quality and arrangement of collagen fibrils, thereby affecting the overall health of the connective tissues. CRISPR/Cas9 offers a viable method for correcting pathogenic genetic variants associated with collagen-related pathologies. This review also emphasizes the need for a deeper understanding of gene-environment interactions, which is critical for the development of personalized medicine for the treatment and management of DDD and related conditions. Expanding genetic studies and integrating insights into biomaterials and tissue engineering are essential for this purpose. A comprehensive genetic approach promises advances in predictive, preventive, and personalized health care.

## 1 INTRODUCTION

Low Back Pain (LBP) is a debilitating condition that affects millions of people globally and requires deeper insight into its molecular basis. LBP remains one of the most prevalent musculoskeletal disorders, ranking among the leading causes of disability and healthcare costs, with over $100 billion spent annually in the United States. This poses a significant global economic burden [1]. The primary driver of LBP is degenerative disc disease (DDD), which has a multifactorial origin and substantial impact worldwide [2, 3]. The intervertebral disc (IVD) provides mechanical stability and flexibility to the spine [4, 5]. However, injury or aging initiates degenerative changes in this weight-bearing structure as early as the second decade of life [6].

IVDs are composed of the Nucleus Pulposus (NP), rich in collagen type II and proteoglycans, and Annulus Fibrosus (AF), predominantly composed of collagen type I. Both interact with and are supported by the Extracellular Matrix (ECM) to maintain disc hydration and resilience [7, 8]. NP resists axial compression through hydrostatic pressure, whereas the AF maintains circumferential integrity by enclosing the NP within the disc structure [9, 10].

The ECM of the disc contains several types of collagen that play essential structural roles in the disc [11]. In degenerative IVD, there is an increase in collagen type I synthesis, whereas the production of types II and IX decreases, contributing to fibrotic changes in the NP. Collagen types IV, V, X, and XI are associated with early disc degeneration, potentially influencing the progression and phenotypic severity of DDD [12-14]. Alterations in the structure and quantity of ECM constituents, such as aggrecan, matrix metalloproteinases (MMPs), collagen, and cytokines, disrupt ECM homeostasis in degenerative IVDs [15]. This results in impaired hydrostatic pressure retention by the NP, decreased disc height, and diminished IVD resistance to compressive forces [9].

A significant number of pathological human diseases have been linked to aberrant collagen gene expression, with 68% of collagen-encoding genes contributing to more than 106 pathological phenotypes. However, the genetic basis of DDD remains unclear. The most commonly mutated amino acid under these conditions is glycine, which is strongly associated with collagen-related disorders [16]. Targeting collagen pathways identified in preclinical models is a promising strategy to improve IVD health and slow the progression of DDD [17, 18]. Moreover, genetic variations in genes critical for IVD growth and stability are linked to DDD, with collagen-encoding genes consistently implicated [9, 19-21]. These genetic variations can increase IVDs' susceptibility to mechanical loading, potentially compromising the AF and affecting disc function [22]. A recent case-control study using whole-genome sequencing identified genetic variants in collagen-encoding genes, with a significant proportion of the cohort harboring mutations, underscoring the importance of these genes in the genetic epidemiology of DDD [23].

DDD has a multivariate etiology, including age, mechanical loading, posture, trauma, and high Body Mass Index (BMI). However, genetic factors play a more substantial role in its pathogenesis than previously recognized, whereas environmental factors have a comparatively minor impact [9, 24-26]. Understanding the mechanisms underlying hereditary influences on DDD pathogenesis is critical for developing targeted interventions [27]. Numerous genetic variants associated with the disease have been identified, particularly those in structural genes encoding collagen, which are pivotal for disc degeneration [28]. These polymorphisms, especially those affecting the development of the collagen triple helix, are known to cause severe connective tissue diseases [29, 30].

The complexity and multifactorial nature of DDD pathogenesis are not fully understood, and the condition continues to impose a significant burden on the global economy due to direct and indirect costs [24, 25]. A deeper understanding of the underlying causes is necessary to delineate the etiology of this disease. The primary role of genetic makeup in disease development suggests that a comprehensive assessment of genetic variations could facilitate early prediction of susceptibility and severity. Moreover, a thorough understanding of hereditary influences could enable physicians to devise more effective therapeutic strategies [27, 28]. This review provides updated insights into the association between genetic polymorphisms in various collagen genes and their roles in DDD pathogenesis. Despite these advances, the genetic basis of DDD remains incompletely characterized, leaving room for investigation into the genotype, disease phenotype, and development of targeted therapies for this condition.

## 2 COLLAGEN TYPE I

Type I collagen accounts for approximately 95% of the collagen in bone and 80% of the total proteins in connective tissues. This type of collagen is found throughout most bones, skin, tendons, ligaments, blood vessels, and the sclera and cornea. Type I collagen consists of two α1 chains encoded by COL1A1 and one α2 chain encoded by COL1A2. It is arranged in fibrils approximately 300 nm in length, 1-5 nm in diameter, with more than 1,000 amino acids in each chain. It contains two non-helical terminal domains, an amino-terminal and carboxy-terminal (N- and C-telopeptides) domains, and a central triple helix domain that comprises approximately 95% of the protein structure [31, 32].

Critical post-translational modifications ensure the attainment of full mechanical functionality and integrity of collagen type I. Disruption at any point in this modification cascade reduces tensile strength and predisposes tissues to degenerative diseases [33]. Recently, degenerative changes and collagen type I formation have been identified as biomarkers of bone and disc degradation, highlighting their potential for clinical assessment [34]. Genetic polymorphisms in COL1A1 influence the biomechanical properties of collagen type I and may compromise the functional integrity of the IVD. Since collagen type I accounts for approximately 70% of the dry weight of AF, genetic variations can result in structural instability and increased sensitivity to mechanical stress [35, 36]. Although these insights have emerged in recent studies, the exact molecular pathways through which COL1A1 polymorphisms influence IVD degeneration remain poorly defined and require further investigation.

### 2.1 COL1A1

COL1A1, which encodes the pro-α1 chain of type I collagen, spans approximately 18,000 base pairs (bp) and includes 52 exons. It is located on chromosome 17 at positions 21.33–22 (17q21.33-q22). This gene has been increasingly recognized as a significant candidate for various disorders, particularly those affecting the musculoskeletal system. Notably, specific single-nucleotide polymorphisms (SNPs) within COL1A1 have been linked to osteoporosis and other musculoskeletal diseases, including DDD [35, 37-40].

The +1245G>T SP1-binding site polymorphism in COL1A1 disrupts the fourth binding site of the transcription factor SP1, resulting in an altered COL1A1/COL1A2 expression ratio. This imbalance destabilizes collagen type I fibers, contributing to IVD degeneration and related disorders, including skeletal connective tissue pathologies. The position of SP1 is shown in Fig. (**1**) [35].

The presence of the T allele at this site leads to increased expression of COL1A1 relative to COL1A2, resulting in an unbalanced COL1A1/COL1A2 expression ratio and subsequently unstable type I collagen fibers. This instability affects the dynamic structure of the AF matrix, influencing DDD and other related conditions [41, 42]. The pathological mechanism driven by SP1 dysregulation is shown in Fig. (**2**). The schematic representation in Fig. (**2**) was partially adapted from previous studies, with the COL1A1/COL1A2 expression derived from [43] and the impaired IVD adapted from [44]. The association between SP1 polymorphism and both DDD and large-joint osteoarthritis (hip and knee OA) has been documented in multiple ethnicities [36].

A study in a Han Chinese population involving 493 patients with DDD reported a statistically significant difference in genotype distribution between cases and controls; the homozygous TT genotype was 8.5% in cases and 2.4% in controls. The TT genotype increased the risk of DDD by approximately threefold. However, the allele frequency was not significantly different between groups; the T allele was present in 19.5% of cases compared to 16% in the controls [45]. Another study involving 36 Greek male soldiers, including 24 with DDD, showed that 33.3% of the DDD patients had the TT genotype, whereas none of the healthy controls did, indicating a significant association (*P* < 0.001) [46].

Moreover, a cohort study in the Netherlands demonstrated that the risk of developing DDD was significantly higher in individuals with the TT genotype than in those with the GT or GG genotypes [36]. However, a preliminary study in an Indian population and a twin study of 588 male monozygotic and dizygotic twins failed to confirm any association between SP1 polymorphisms and different DDD phenotypes; the minor T allele frequency was 14% and 12% in cases and controls, respectively [29, 35]. Moreover, studies in Turkish populations suggest that the SP1 allele may be associated with more severe DDD. [47]. A meta-analysis of seven studies supported the association between SP1 polymorphisms and DDD and other musculoskeletal disorders [48]. While the association between SP1 and DDD susceptibility has been replicated in several ethnic cohorts, including Chinese, Greek, Dutch, and Turkish populations, investigations in Indian and Finnish cohorts have failed to replicate this correlation. This conflict likely resulted from several methodological and population-specific differences, including the study design and sample size. The Indian study employed stricter inclusion criteria and involved a relatively small sample size, which may have limited its ability to detect significant associations between variables. In contrast, the Finnish study recruited twins, a sample with less genetic heterogeneity, potentially limiting the generalizability of the findings to the broader population and diminishing the observable impact of individual genetic variants, such as SP1.

Additionally, another SNP in COL1A1, rs2075555, which involves the replacement of adenine (A) with cytosine (C) at the 9710 position (9710A>C), has been associated with disc signal intensity, a primary phenotype of DDD representing intradiscal water content within the disc (Fig. **3**) [49]. However, a Norwegian case-control study reported no association between rs2075555 and DDD [50]. Although both study populations were Caucasian, the inconsistency in the association between the rs2075555 polymorphism and DDD may be attributed to key methodological and demographic differences. The Finnish study included only male monozygotic and dizygotic twins, providing a well-controlled cohort. In contrast, the Norwegian study involved a heterogeneous clinical sample of both men and women with varying durations and severities of LBP and included patients undergoing different treatment modalities. Sex differences may also contribute to conflicting findings, as sex-specific genetic effects have been observed in musculoskeletal traits, and hormonal influences can modulate gene expression or phenotype penetrance differently in males and females. Collectively, these disparities in study design, phenotyping methods, and population structure likely explain the lack of replication in the Norwegian cohort study.

A case-control study in an Iranian cohort investigated another SNP, rs909102, which involves the substitution of Guanine by Thymine at position 50184005, as depicted in Fig. (**4**). They concluded that in patients with DDD, the T allele of rs909102 was 1.17 times more prevalent than in controls, although the difference was not statistically significant [12]. Their findings are consistent with those of an earlier Twin Spine study, which indicated that rs909102 was associated with reduced signal intensity in the lower lumbar discs, although this association was not significant [29]. Table **1** summarizes the COL1A1 SNPs with reported or potential associations with DDD.

Despite these advances, the molecular pathways linking ECM degradation to genetic variations remain poorly defined. Future studies are needed to clarify these mechanisms and identify therapeutic targets to halt or reverse ECM degradation in DDD.

## 3 COLLAGEN TYPE II

Type II collagen is a fibrillar collagen subtype that is mainly distributed in cartilage and forms a dense network that contributes to the tensile strength and elasticity of the cartilage. In IVDs, its expression predominantly occurs within the NP and is synthesized by chondrocyte-like cells. This collagen type is essential for maintaining the biomechanical integrity and hydration of the NP, enabling it to resist compressive force. Abnormalities in collagen type II fibrils, encoded by the COL2A1 gene, have been implicated in a diverse range of disorders, including DDD [51-53].

### 3.1 COL2A1

COL2A1, located at chromosome 12 positions 13.11–13.2, encodes the α1 chains of homotrimer collagen type II [54]. Variants of this gene disrupt normal collagen synthesis, including the integrity of the triple-helical structure [55]. Mutation update for COL2A1 gene variants associated with type II collagenopathies. Human Mutation, 37(1), 7-15). This has resulted in a multitude of autosomal disorders, such as achondrogenesis type II, Kniest dysplasia, Stickler syndrome type I, spondyloepiphyseal dysplasia congenita, and knee OA. Mutations and SNPs in COL2A1 are essential for disease susceptibility within the framework of DDD [51].

Recently, research has focused on the involvement of SNPs in COL2A1 DDD. For example, the rs1793953 SNP, located in the COL2A1 intron (G/A), is a protective factor for OA. Deng *et al.* [56] found that A-allele carriers have a diminished risk of DDD, proving that this is an important genetic factor. The location of this SNP is illustrated in Fig. (**5**). The rs2276454 SNP in exon 34 of COL2A1 results in a T/C substitution at position 47999758 and is associated with DDD. Deng *et al.* [56] noted that compared to carriers of the T allele, carriers of the C allele had an increased risk of DDD at an estimated rate of 1.8-fold. A case-control study further supported this association, with carriers of the C allele accounting for 54.1% of cases compared to 39.5% in controls, and carriers of the C allele having a 1.75-fold higher risk than carriers of the T allele. Consistent findings were reported in a case-control study investigating the association between rs2276454 and DDD pathology, which found a significant difference in the distribution of the C allele between cases (53.9%) and controls (40%); the C allele increased the risk by 1.75 times [57]. Moreover, a case-control study in the Korean population investigated the association between rs2276454 and Degenerative Lumbar Scoliosis (DLS). The study reported that this SNP increases the risk of DLS by 1.90-fold and 3.58-fold in the co-dominant and dominant models, respectively [54].

Other COL2A1 SNPs have also been investigated, including rs1793937, an alternatively spliced exon two variant. Deng *et al.* [56] found no significant association between DDD and this SNP, showing similar distributions of wild-type and variant alleles in cases and controls. Another missense substitution, rs2070739, which was represented as a C/T substitution, was also not significantly associated with DDD. The distribution of the wild-type allele was 49% in the cases and 50.9% in the controls, whereas that of the variant alleles was 51% in the cases and 49.1% in the controls [56]. Another SNP, rs2070739, represents a missense variant characterized by a C/T substitution in COL2A1, which converts codon 1405 from GGC (glycine) to AGC (serine), as shown in Fig. (**6**). Li *et al.* [57] examined the association between rs2070739 and DDD etiology; however, they reported no significant association between this SNP and DDD [58]. To the best of our knowledge, the consequences of replacing glycine (non-polar) with serine (polar) in collagen II structure and function must be further investigated using predictive modelling. These findings are summarized in Table **2**, which lists the COL2A1 SNPs that may be associated with DDD.

Only the rs2276454 variant has been replicated in three independent studies, whereas the other COL2A1 SNPs have not been investigated in different ethnic groups. Therefore, the absence of replication should not be interpreted as a lack of association but rather underscores the need for further studies across diverse populations to confirm or refute the potential role of these SNPs in DDD susceptibility. This manuscript describes the COL2A1 polymorphisms associated with DDD. Furthermore, the exact molecular mechanisms underlying these genetic associations remain unclear; therefore, it is important that future studies elucidate how these SNPs may modulate collagen fibril assembly to identify potential targets for intervention in IVD.

## 4 COLLAGEN TYPE IX

Collagen type IX is a member of the FACIT family, which includes three chains, α1, α2, and α3, encoded by COL9A1, COL9A2, and COL9A3, respectively. Its general molecular structure consists of three collagenous domains (COL1, COL2, and COL3) alternating with four non-collagenous domains (NC1-NC4). This triple helical structure is stable because of disulfide bonds, which allow collagen IX to bind to the ECM and enhance its stability. Additionally, the COL2 domain of collagen IX forms covalent crosslinks with type II collagen, strengthening the integration of collagenous and noncollagenous elements within the ECM [59-61].

Collagen IX is present in small amounts in the NP and AF of the IVD and plays a crucial role in maintaining the structural integrity of these regions [62, 63]. Beyond the IVD, collagen IX significantly contributes to the stability of the fibrillar collagen network in the cartilage matrix of the articular cartilage. It also anchors proteins, such as matrilin-3 and proteoglycans, regulating the diameter of collagen fibrils and maintaining the functional properties of the articular cartilage [64]. The essential roles of collagen IX in the IVD and articular cartilage underscore the importance of COL9A1, COL9A2, and COL9A3 as candidate genes in DDD [62].

Studies have demonstrated that Polymorphisms in COL9A2 and COL9A3 are associated with an increased risk of DDD onset and progression [9]. Matsui *et al.* [65] reported that tryptophan (Trp) polymorphisms in COL9A2 and/or COL9A3 are significantly associated with spinal stenosis with spondylolisthesis, a specific DDD phenotype. The presence of Trp alleles in these genes increased the risk of developing this condition by approximately 6.81-fold. This genetic link highlights the potential of targeted genetic studies and interventions for the management and understanding of DDD and its associated conditions.

### 4.1 COL9A2

COL9A2 encodes the α2 chain of collagen IX and is located on chromosome 1 at 1p34.2 It consists of 32 exons spread over 15,000 base pairs and plays a crucial role in ECM integrity in the IVD and cartilage [66, 67]. Deletion of COL9A2 in transgenic mice accelerates DDD progression [68]. Additionally, several studies have identified COL9A2 genetic polymorphisms associated with an increased risk of DDD onset and progression [9, 69].

Annunen *et al.* [59] initially reported a COL9A2 polymorphism associated with DDD. rs120077871 represents a base substitution in exon 19, where thymine replaces cytosine, causing tryptophan substitution in the Trp2 allele. This variant is strongly associated with DDD in the Finnish population, and subsequent studies have supported this association in age-dependent and severity-specific contexts. They analyzed sequence variations and identified a single nucleotide polymorphism (SNP) that substitutes tryptophan (Trp) for glutamine (Gln) or arginine (Arg) at position 326 (Gln326 or Arg326 Trp). In their study, six of 157 patients with DDD carried the Trp2 allele, whereas none of the 174 controls did. They used family-based pedigree analysis to confirm that the Trp2 allele was disease-causing, given the small population size.

The Trp2 allele (rs120077871) in COL9A2 involves a base substitution at exon 19, where the first cytosine in codon 326 (CGG coding for Arg) is replaced by thymine (TGG coding for Trp), as illustrated in Fig. (**7A**). Further studies in other populations have explored the role of this allele. For instance, a German study reported the Trp2 allele in 1.3% of the cohort, suggesting that it is a potential age-dependent risk factor [70]. Additional studies have suggested that the Trp2 allele is associated with disease severity in older individuals, indicating a significant correlation with disc prolapse in certain patients [71].

The Trp2 allele has emerged as an age-dependent risk factor in Han Chinese individuals, where the Trp2 allele represents 19.9% of the population. It was also associated with a 4-fold increase in the risk of annular tears at 30–39 years of age and a 2.4-fold increase in the risk of DDD and endplate herniations at 40–49 years of age [72]. Consistently, German [70] and Indian [73] studies have identified its role in disease severity, whereas Japanese [74] and Southern European studies [47, 75] have failed to establish any significant association. The prevalence of Trp2 was 11.59%, 1.7%, and absent in the cohorts of Japanese, Turkish, and Greek populations, respectively. This finding demonstrates ethnic variation in allele frequency and genetic risk. These findings suggest that the presence of the Trp2 allele exacerbates DDD severity in this age group, with a higher prevalence of Trp2 alleles than that in the Finnish population [72]. A study involving patients with scoliosis demonstrated that the Trp2 allele affects the mechanical properties of the IVD. The presence of Trp2 reduced the swelling pressure and compressive modulus of the NP, impairing the triple-helical structure of collagen IX and, consequently, disrupting its interactions with collagen II and proteoglycans. This disruption leads to a significant deterioration in the disc integrity, as shown in Fig. (**8**) [76]. In Fig. (**8**), the collagen IX structure was adapted from [77], the IVD under swelling pressure was adapted from [78], and the degenerated IVD representation was adapted from [79].

Moreover, a case-control study enrolling 200 individuals demonstrated that the Trp2 allele increased the risk of DDD by 2.39 times and was significantly associated with disease severity [73]. Higashino *et al.* [80] found that patients under 40 years of age harboring the Trp2 allele exhibited higher DDD severity, suggesting a six-fold increase in disease severity in this age cohort. They considered Trp2 to be an age-dependent risk factor. Furthermore, a study in an Indian population demonstrated that Trp2 is a significant risk factor for DDD onset, especially in heterozygous individuals [81].

Conversely, studies in the Japanese population and among Southern European ancestors have reported no significant association between the Trp2 allele and DDD etiology, despite having larger sample sizes than those in the Finnish study. These studies have highlighted the variability in allele distribution and its association with DDD across different populations [47, 74]. Recently, a case-control study in Iranian patients with DDD also reported no association between the Trp2 allele and DDD; the Trp2 allele was present in 22.8% of cases and 22.9% of controls [12]. Consistent with these results, Bageri *et al.* [82] reported no association between Trp2 and DDD phenotypes in a Southwestern Iranian population. A pilot observational study conducted among Chinese Singaporeans reported similar findings [83]. Nevertheless, the association between Trp2 and DDD was supported by a meta-analysis that included 15 studies, 292 cases, and 2089 controls. The Trp2 allele increased the risk of DDD by 1.43 in all recruited ethnicities, although this association was not statistically significant (*P* = 0.058). Conversely, in the Caucasian subgroup, the association was significant (*P* = 0.022), with the Trp2 allele increasing the risk by 2.62 times [84].

Furthermore, exon 19 of COL9A2 has another notable nucleotide substitution, replacing the second adenine nucleotide in codon 326 (CAG coding for Gln) with guanine (CGG coding for Arg), as shown in Fig. (**7A**) [59]. The Arg2 allele has a high frequency across populations, and a study of German patients found a potential association with DDD etiology when homozygous for the Arg2 allele. However, this association was not statistically significant [85]. Consistent with these findings, Meng *et al.* [86] reported that homozygosity for Arg increased the risk of DDD by 2-fold. In contrast, studies from Greece [75], Japan [74], and Finland [59] have reported no association between Arg and DDD pathophysiology. Table **3** summarizes the findings for Trp2 and Arg2 in relation to DDD.

Although the Trp2 allele in COL9A2 has been documented to increase DDD in different studies, the overall findings across populations have remained inconsistent. While early investigations, such as the original Finnish study [59], reported a robust association between Trp2 and DDD using a small but well-characterized cohort, other studies, particularly those from Japanese [74], Iranian [12, 82], Turkish [47], Greek [75], and Chinese-Singaporean [83] populations, failed to replicate this association. Although ethnic differences and allele frequencies likely contribute to some extent, they do not fully explain the conflicting results, especially given that two independent Japanese studies reported opposing outcomes: one identified a significant association [80], while the other found no association [74]. These discrepancies may be attributed to variations in study design, methodological approaches, and phenotype definitions.

One major source of inconsistency is the heterogeneity of the diagnostic criteria and grading systems used to define DDD. Some studies have focused on clearly defined and clinically significant phenotypes, such as disc herniation, annular tears, and Schmorl’s nodes, using standardized MRI-based grading systems such as Schneiderman’s classification. In contrast, others have used broader or modified definitions that encompass a wider range of degenerative changes, including subclinical or age-related findings. Moreover, the absence of Trp2 or low allele frequencies in particular cohorts (*e.g*., Turkish [47], Greek [75], and German [85]) further limits the statistical power to detect meaningful associations and increases the likelihood of type II errors, rather than confirming a genuine absence of effect. In some studies conducted in Southern China [72], Japan [80], and Germany [70], control groups were not included, making it difficult to determine whether the observed frequency of Trp2 in DDD patients represents a genuine overrepresentation or reflects its baseline prevalence in the general population. The lack of controls or the use of poorly matched or undefined comparator groups introduces interpretative challenges and undermines the strength of any observed associations.

Importantly, several studies that reported a significant association highlighted that Trp2 may act as an age- or severity-dependent modulator. For instance, both Chinese [72] and Indian [73] studies found that the Trp2 allele was more strongly associated with early onset and severe forms of DDD, respectively. This suggests that its pathological impact may only emerge in certain subgroups rather than in the general population. Studies that did not incorporate age stratification or failed to focus on severe phenotypes may have overlooked these effects. Furthermore, inconsistencies in genotyping methods, ranging from PCR with sequencing and restriction fragment length polymorphism (RFLP) to allelic discrimination *via* real-time PCR, can introduce technical variability, particularly when different studies refer to rs120077871 and rs2228564 interchangeably, despite both affecting codon 326 but referencing different wild-type alleles (Arg or Gln).

Taken together, these findings suggest that the contribution of the Trp2 allele to DDD is likely context-dependent and shaped by genetic background, methodological rigor, and phenotypic stratification. Rather than being a universally applicable risk variant, Trp2 may represent a susceptibility allele with variable penetrance that becomes pathogenic under certain biological or environmental conditions. To resolve these discrepancies, future studies should adopt standardized diagnostic criteria, clearly defined phenotypes, adequate age stratification, and large ethnically diverse cohorts using uniform genotyping platforms. Only through such harmonized approaches can the true significance of Trp2 in DDD be definitively established 

Additionally, an SNP at codon 335 substitutes the first cytosine (CTA, coding for leucine) with guanine (GTA, coding for valine), known as the Val2 allele (Fig. **7B**) [87]. One study reported that Val2 is a protective allele against severe DDD [83]. Consistently, another study in a Chinese population reported a higher Val2 allele frequency among controls (34.1%) than among cases (26%) [88]. Conversely, studies in the Japanese [74] and Finnish [87] populations reported no significant association, although the frequency of the Val2 allele in the Japanese population was slightly higher in the control cohort than in the cases. Nevertheless, a meta-analysis found that it is associated with lumbar DDD in Asian populations, emphasizing the complex genetic landscape of DDD [89]. Table **4** highlights research on Val2 in relation to the DDD.

Despite some inconsistencies across studies, significant associations between specific haplotypes in COL9A2 and DDD have been identified, including the “221” haplotype comprising rs209923, rs7533552 (Arg2 allele), and rs2076696, which was predominantly found in cases and was significantly associated with DDD etiology [74].

### 4.2 COL9A3

*COL9A3* encodes the collagen IX α3 chain and is located on chromosome 20 at q13.3 It contains 32 exons spanning about 23,000 base pairs. COL9A3 is an essential gene for ECM integrity and has been associated with several age-related diseases, such as DDD and OA [90, 91]. COL9A3 has been identified as a predisposing factor for several age-related disorders, including OA and DDD [92, 93]. Recent studies have highlighted a significant role of COL9A3 in disease pathogenesis. For example, silencing COL9A3 in the NP of rats with DDD was found to induce apoptosis *via* activation of the MAPK signaling pathway, whereas stimulating its expression suppressed apoptosis, thereby demonstrating its crucial role [94].

Research has indicated that Several variants of COL9A3 have been associated with disease etiology [9]. Trp3 SNP (rs61734651) in exon 5 of COL9A3: Arg to Trp substitution at codon 103. It has been associated with a threefold increased risk of DDD in Finnish populations, particularly in obesity/high BMI subjects, showing complex gene-environment interactions. This Trp3 allele was found in 12.2% of Finnish DDD cases, compared with 4.7% in the control group, significantly increasing the risk by 3-fold in heterozygous individuals [87]. Additionally, the presence of the Trp3 allele exacerbates the effects of elevated BMI on DDD risk. Obese individuals with the Trp3 allele are more likely to develop DDD [95]. The impact of this allele on DDD is moderated by the presence of the IL-1betaT (3954) polymorphism; without it, the risk of developing dark NP increases sevenfold, highlighting the complex gene-gene and gene-environment interactions involving COL9A3 [96]. Furthermore, males carrying the Trp3 allele have a 5.83-fold increased risk of developing DDD, underscoring the need for further studies to elucidate the underlying mechanisms [82].

The association of the Trp3 polymorphism with DDD has been reported to show significant variation across populations, with strong links observed in Finnish [87], American [65], and Turkish [47] cohorts. At the same time, studies have shown no such significant link in Singaporean [83], Greek [75], and Indian [81] populations, thus emphasizing the genetic and environmental complexity of DDD susceptibility. For example, a case-control study in Singapore found that the Trp3 allele was absent in both patients and controls [83]. Similar findings were reported in Greece, where 4.3% of individuals carry Trp3, suggesting no significant link between Trp3 and DDD etiology in this population, despite its geographic proximity to Turkey [47, 75]. In India, the Trp3 allele is less likely to be a risk factor for DDD [81]. These findings are further supported by research conducted on young Finnish adults [22], as well as a study of 12-14-year-old Danish children [97], both of which concluded a lack of association between Trp3 and DDD genetic epidemiology, and a meta-analysis that failed to find a correlation between Trp3 and lumbar DDD in Caucasian populations [89]. Additionally, another meta-analysis of 13 studies, including 1623 cases and 1606 controls, found no association [84]. Table **5** summarizes studies on Trp3 and Arg3 and their potential association with DDD.

The conflicting evidence regarding the association of the Trp3 allele (rs61734651) in *COL9A3* with DDD across different studies is unlikely to be explained by a single factor. Instead, it reflects a combination of biological complexity and methodological variability that hinders consistent replication. One crucial but often overlooked factor is the role of gene–gene interactions. Solovieva *et al.* [96] reported that the Trp3 allele was associated with DDD primarily in the absence of the *IL-1β* (C3954–T) polymorphism, suggesting that the genetic risk conferred by Trp3 may depend on the presence or absence of specific alleles in other inflammatory pathways. Interestingly, a later study by Eskola *et al.* [97], also conducted in a Danish cohort, did not confirm this interaction, highlighting the challenges of reproducibility even within similar populations. Notably, this gene–gene interaction has not been investigated in most other studies examining Trp3, limiting the ability to fully evaluate its contribution to DDD susceptibility.

Another source of inconsistency is gender-specific effects. Several studies that reported a significant association between Trp3 and DDD included only male participants, such as those by Solovieva [95, 96], Toktaş *et al.* [47], and Kales *et al.* [75]. In contrast, other studies either included mixed-gender cohorts or did not stratify by sex, which could obscure a possible gene–gender interaction. Supporting this hypothesis, Bagheri *et al.* [82] observed that the association between Trp3 and DDD was evident specifically in men. This inconsistency in gender representation across studies undermines standardization and complicates the interpretation and generalization of results.

Ethnic differences also play a role, particularly in the distribution of the Trp3 allele. For example, Lim *et al.* [83] reported a complete absence of the allele in both patients and controls in a Chinese Singaporean cohort. This finding indicates that the lack of association in some populations may reflect the true absence of the variant rather than a lack of biological effect. However, contrasting findings between geographically proximate populations, such as Turkey, where an association was found, and Greece, where it was not, suggest that additional factors beyond ethnicity, such as lifestyle, environmental exposures, or population-specific gene–environment interactions, may influence the penetrance of Trp3.

Variation in phenotyping methods further complicates interpretation. Studies differed in how DDD was defined and graded; some used the Schneiderman or Pfirrmann classification systems, while others employed modified or multilevel scoring schemes. This lack of uniformity in diagnostic criteria introduces the possibility of phenotype misclassification, reducing the likelihood of detecting true genetic effects. Additionally, the timing of the assessment may influence outcomes. Studies involving younger populations, such as the Danish [97] and Finnish [22] cohorts of adolescents and young adults, did not observe any association between Trp3 and DDD. While this may seem to indicate a null finding, it is more likely that the degenerative changes associated with Trp3 require time to manifest and may not be detectable during early life stages. The absence of longitudinal data in these studies prevents assessment of whether Trp3-related degeneration might emerge later in adulthood, underscoring the need for long-term follow-up in future genetic epidemiology studies.

Overall, the inconsistencies in Trp3 findings cannot be attributed to a single explanation but rather reflect a complex interplay of gene–gene interactions, sex-specific effects, ethnic variation in allele frequency, methodological heterogeneity, and cohort characteristics such as age and duration of follow-up. Although Trp3 has shown significant associations with DDD in several populations, its role remains context-dependent. To clarify its contribution to DDD, future research should adopt standardized diagnostic frameworks, investigate potential interactions with other genetic and environmental factors, and utilize large, diverse, longitudinally followed cohorts. Only through such comprehensive approaches can the biological relevance of Trp3 in DDD be fully elucidated.

Recently, a study in the Chinese Han population identified an intronic polymorphism (rs6122316) in COL9A3 that substitutes T with C at position 61,447,759. This polymorphism was significantly associated with lumbar disc herniation (LDH) in individuals aged ≥ 50 years, as assessed using codominant and recessive models [98]. Additionally, pedigree-based analysis of a young boy with early onset, multi-segment LDH revealed a disease-causing SNP in COL9A3, changing Arg-384 to Trp (Fig. **9A**). Predictive modeling suggested that this substitution affects protein stability, charge properties, and spatial conformation, likely altering the lysyl oxidase-catalyzed crosslinking process and thereby reducing disc stability, which could precipitate the onset of degenerative processes in the IVD (Fig. **10**, where the collagen IX structure was adapted from [77]) [99]. While these findings provide compelling evidence for the involvement of rs6122316 and the Arg-384 variant in LDH susceptibility, both polymorphisms require replication in independent, ethnically diverse populations to confirm their generalizability and to establish their relevance across different genetic backgrounds. Moreover, future studies should investigate whether these SNPs are explicitly associated with LDH or whether they also contribute to other DDD phenotypes. The detailed codon substitutions in COL9A3 are summarized in Fig. (**9B**).

## 5 COLLAGEN TYPE XI

Collagen type XI consists of a heterotrimer of three chains, α1(XI), α2(XI), and α3(XI), encoded by COL11A1 and COL11A2, with variable expression of COL11A3. It is considered an important component of cartilage ECM and IVD ECM, where it organizes and crosslinks collagen fibrils (particularly types II and IX) and plays a structural role. Its presence is vital for maintaining cartilage integrity and IVD stability. This type of collagen is specific to cartilage and plays a crucial role in organizing the ECM by participating in collagen fibril formation. Collagen XI aids in the maturation of cartilage collagen molecules by crosslinking with collagen types II and IX, thereby enhancing their structural integrity and stability [100, 101].

Structurally, collagen XI resembles collagen types I, II, and III in terms of molecular size. It is imperative to regulate the spacing and diameter of collagen I and II fibrils. A reduction in collagen XI levels can lead to an abnormal fibril structure, with alterations in the regulation of fibril diameter, resulting in decreased diameter and disrupted fibril alignment. These structural anomalies are critical because collagen XI deficiency is associated with chondrodystrophy in both mice and humans, highlighting its essential role in skeletal development and maintenance. Moreover, mutations in collagen XI have been linked to increased degradation of collagen II, further underscoring its importance in maintaining cartilage integrity [100, 102].

It is expressed in various tissues, including cartilage and skeletal muscle, and notably in both the AF and NP of the IVD. Collagen XI also plays a pivotal role in linking proteoglycan aggregates to the collagen fibrillar meshwork in cartilage. This connection is crucial for anchoring cartilage proteoglycans, which are essential for maintaining the gel-like nature and mechanical properties of cartilage [103].

Mutations in human collagen XI have been reported to cause type XI collagenopathies, which are characterized by vertebral abnormalities and disc herniation. These findings highlight the significant role of collagen XI in maintaining IVD structural integrity. Given its essential functions in the IVD and interactions with other collagen types, collagen XI is considered a critical factor in the onset and progression of DDD [103].

### 5.1 COL11A1

COL11A1, located on chromosome 1 at 1p21.1, encodes the α1 chain of type XI collagen. This gene contains 68 exons and spans approximately 250,000 bp. It is crucial for collagen fibril assembly, as its downregulation results in abnormalities in fibril diameter and misalignment, thereby weakening cartilage integrity [104, 105], This gene plays a crucial role in the production and assembly of collagen fibrils. Silencing it significantly impairs the formation of these structures, thereby reducing the mechanical stability of articular cartilage through collagen XI depletion. This deficiency increases collagen II's susceptibility to mechanical degradation [106]. An *in vivo* study using transmission electron microscopy showed that COL11A1 deficiency leads to abnormal collagen fibrils with reduced diameters and misalignment, underscoring its role in maintaining proper collagen structure [107].

Research has shown that a frameshift polymorphism in COL11A1 can lead to chondrodysplasia in mice, highlighting its essential role in skeletal morphogenesis [108]. SNP rs1676486 in exon 62 of COL11A1 was significantly associated with both disc herniation and DDD. This missense mutation replaced proline with serine at position 1535. The Ser allele increases the risk of LDH by 1.5-fold. Asian studies have reported a stronger association: homozygous carriers have a 2.28-fold increased risk of CDD. The Ser allele was more frequent in the case group (35%) than in the control group (26%), suggesting a potential role in DDD pathology. Additionally, as DDD severity increases, COL11A1 expression in the IVD decreases, suggesting that the Ser allele may influence COL11A1 transcripts [103].

A larger case-control study involving 18,000 individuals reported a strong association between the Ser allele and LDH, as well as an allelic expression imbalance in COL11A1. The presence of the Ser allele is associated with reduced gene expression [109]. Furthermore, research in the Chinese population corroborated these findings, reporting that 10.51% of cases were TT homozygous compared to 7.33% in controls; the Ser allele increased the risk of LDH by 1.36-fold and was significantly associated with increased disc degeneration severity in homozygous patients [110]. In a Japanese population, this SNP also showed clinical relevance but in a different anatomical context: among collegiate male wrestlers, the Ser allele was significantly associated with cervical DDD, with homozygous individuals exhibiting a 2.28-fold increased risk. The allele frequency of the Ser variant in the cervical DDD group was 40.0%, compared with 25.5% in controls, indicating a notable increased risk. However, in the lumbar group, the Ser allele was present in 31.5% of cases and 34.1% of controls, showing no significant difference [111]. This difference may reflect differences in the contributions of genetic and environmental factors at different spinal levels. A classical twin study involving 326 monozygotic and dizygotic twins quantified the heritability of DDD using MRI-based phenotyping and found that genetic influence was greater in the cervical spine (79%) than in the lumbar spine (64%) when focusing on severe degeneration scores. These findings suggest that cervical degeneration is more strongly driven by genetic factors, whereas lumbar degeneration is comparatively more influenced by environmental and mechanical factors such as occupational strain and lifestyle [112]. In addition, Japanese athletes showed a significant association between this SNP and the limbus vertebra [113]. Table **6** provides an overview of the impact of rs1676486 on DDD across different studies.

A meta-analysis of four studies concluded that the Ser allele is a risk factor for DDD in Asian populations [114]. Xie and colleagues, in another meta-analysis, suggested that this polymorphism is associated with DDD in a dose-dependent manner, with homozygotes at higher risk than heterozygotes. They proposed that modulating this locus might help prevent the onset and development of DDD [84].

Videman *et al.* [29] reported another SNP in COL11A1, rs1337185, with a minor allele frequency of 0.28%. They found an association between this variant and disc bulging using QTDT-based analysis, indicating its relevance in DDD pathology. This intronic SNP, positioned at chr1:103079209, has also been linked to an increased risk of disc herniation, spinal stenosis, or spondylolisthesis in a Chinese Han population study, with significant risk increases of 1.8-fold and 1.92-fold, respectively [115, 116]. However, Rajasekaran *et al.* [27, 117] reported mixed results across studies, with significant associations for severe Total Disc Degenerative Score (TDDS) in one study but no correlation with other DDD pathological phenotypes in another. These findings underscore the complexity of DDD pathogenesis and the critical role of genetic factors, including those involving COL11A1. Table **7** summarizes the association between rs1337185 and DDD across studies.

### 5.2 COL11A2

COL11A2 is located on chromosome 6 at position 6p21.32, which encodes the α2 chain of collagen XI, with a total of 72 exons. It is a critical gene involved in skeletal development. Variants of COL11A2 are associated with early-onset DDD-related OA and DDD [118, 119]. This gene is crucial for vertebral development and has been implicated in various skeletal and connective tissue disorders. Researchers using CRISPR/Cas9 technology have introduced a nonsense mutation that truncates α2 chain synthesis at amino acid 642 and completely deletes the gene in zebrafish. Both genetic modifications resulted in vertebral malformations, particularly caudal spine fusions, attributed to IVD mineralization [120]. Further studies in zebrafish have shown that silencing COL11A2 leads to severe, early-onset OA, linked to alterations in cell behavior that predispose to joint pathologies [121].

In the context of bone development, COL11A2 is expressed in immature osteoblasts, indicating its involvement in early stages of bone formation [122]. Genetic variants of COL11A2 have been associated with several disorders, including Stickler syndrome [123], Kawasaki disease [124], fibrochondrogenesis [125], and DDD [126].

According to Xu *et al.* [127], a COL11A2 variant, rs9277935, is associated with DDH and increased COL11A2 expression. Other polymorphisms, including IVS6-4A>T, which are associated with lumbar spinal stenosis (LSS) and ossification of the posterior longitudinal ligament (OPLL), significantly increase the risk of disease in susceptible individuals. This variant not only increased COL11A2 expression but also appeared to regulate its chondrogenic properties *in vitro*. Additionally, OPLL, a major contributor to spinal stenosis, has been associated with a COL11A2 polymorphism, IVS6–4A>T, in intron 6. This polymorphism leads to skipping of exon 6, which affects the normal splicing process of the gene [128]. A case-control study revealed a strong association between this variant and LSS, with a prevalence of 93.1% in the case group and 72.3% in the control group [129]. Nevertheless, the influence of this SNP is consistent across studies, as demonstrated by a study of Finnish [130] and Danish [97] Populations that reported no association between the variant and disease etiology. To the best of our knowledge, this inconsistency of IVS6–4A>T is multicausal. Differences in phenotype definition and diagnosis appear to be a significant contributor. The initial Finnish study by Noponen-Hietala *et al.* [129] examined clinically and radiologically confirmed cases of LSS, using high-resolution MRI and CT scans to assess structural features. In contrast, the later Finnish study by Virtanen *et al.* [130] relied primarily on self-reported symptoms and latent class analysis to define degenerative phenotypes, without direct imaging-based confirmation. Similarly, the Danish study by Eskola *et al.* [97] focused on early imaging changes in a young, general population cohort aged 12–14 years. At this stage of life, several structural manifestations influenced by genetic predisposition may not yet be visible, potentially masking any association with the IVS6–4A>T variant.

Additional differences in imaging modality may also account for variation in findings. For example, Eskola *et al.* [97] used a low-field 0.2T MRI, which may have limited sensitivity for detecting subtle structural abnormalities, especially when compared to the 1.5T MRI and CT imaging used in earlier studies. Moreover, the Finnish cohort in Virtanen’s [130] study was occupationally defined and included individuals exposed to long-term whole-body vibration, an environmental factor that could either overshadow or interact with genetic predisposition in complex ways. Age and disease stage also play a significant role. The two studies that failed to identify an association included younger individuals or middle-aged workers, in whom degenerative changes may be less advanced or driven more by mechanical factors than genetic ones. In contrast, both Maeda’s Japanese cohort [128] and Noponen-Hietala’s Finnish patients [129] were older and clinically symptomatic, providing a better opportunity to detect genotype-phenotype correlations. These findings suggest that the inconsistent results surrounding the IVS6–4A>T polymorphism are likely due to a combination of study design, participant age, phenotyping criteria, imaging sensitivity, and environmental exposures, rather than to ethnic variation alone. Understanding the role of this polymorphism in spinal degeneration will therefore require more standardized approaches, age-stratified analyses, and careful consideration of environmental confounders in future studies.

Solovieva *et al.* [96] identified another sequence variation in intron 9 of COL11A2 by replacing adenine (A) with guanine (G). The G allele is significantly associated with disc bulges and increases the risk of such conditions 2.1-fold. Research conducted in Finland indicated that the polymorphisms rs2072915, rs9277933, and rs2076311 are significantly associated with a marked reduction in disc signal intensity, suggesting that these variants influence disc health [29]. However, another meta-analysis reported no association between rs2076311 and DDD [131].

This variant, rs2071025, conferred a 1.47-fold increased risk of DDD in the Chinese Han population. Another variant, rs986522, has been identified as a sex-dependent risk factor in women. However, no significant association of these variants was found in studies within Finnish populations. This study also identified rs986522 as a sex-dependent risk factor, particularly in a female cohort. Additionally, under dominant and log-additive genetic models, rs17214944 was found to increase the risk of DDD by 1.56-fold and 1.6-fold, respectively [126]. Table **8** summarizes the COL11A2 variants and their association with DDD. These findings highlight the complex genetic landscape of COL11A2 and its pivotal role in spinal health and diseases. The involvement of COL11A2 variants in DDD has not been elucidated. Thus, future studies should focus on the functional analysis of these SNPs, with particular emphasis on their implications for collagen XI fibril assembly in IVDs.

## 6 ADVANCED GENETIC THERAPIES

The enormous advancements in gene editing and RNA-based therapies have opened promising and vital avenues for treating DDD by targeting multiple signaling pathways, including collagen-related pathways. These strategies aim not only to prevent further ECM degradation but also to restore the structural integrity and biomechanical function of the IVD. A study using a rat model with coccygeal DDD demonstrated that delivering mRNA encoding the cartilage-anabolic transcription factor (RUNX1) can significantly increase IVD height compared to control and also enhance water content. Notably, this approach shows remarkable increases in collagen II gene expression, along with other ECM components [132]. Importantly, RNX1 has been documented to govern collagen II expression. These findings were also supported by another study that administered Runx1 mRNA directly to damaged discs in a rat model, thereby alleviating the hemostasis imbalance in the ECM and leading to increased disc height, reduced disc space narrowing, and enhanced ECM regeneration [133]. These critical insights indicate the tremendous efficiency of using both CRISPR/Cas9 and RNA-based approaches to treat DDD by targeting either collagen genes or genes with key functions in collagen fiber assembly, quantity, and diameter.

### 6.1 CRISPR/Cas9

CRISPR/Cas9 is a pioneering DNA-editing technology that enables accurate, efficient, and cost-effective modification of genetic material within living organisms to a specific location in the genome. In the context of DDD, CRISPR/Cas9 not only offers promising avenues for therapeutic intervention but can also enhance our understanding of the involvement of several candidate genes, including collagen-encoding genes, in DDD pathogenesis [134]. Recently, Seidl *et al.* [135] applied CRISPR/Cas9 to modulate the ECM in age-related degeneration. The study used Cas9 ribonucleoproteins to knock down the collagen-degrading enzyme MMP-13 in human osteoarthritic chondrocytes. This significantly reduced MMP-13 protein and enhanced type II collagen accumulation in 3D cartilage cultures. These findings indicate that CRISPR/Cas9 can alter gene expression in a population of human chondrocytes. Since IVD has similar chondrocytes and ECM components, these insights give potential to use CRISPR/Cas9 to restore the normal balance of ECM in degenerative IVD. An *in vitro* study that delivered lentiviral CRISPR tools into degenerative IVD cells to inhibit tumor necrosis factor-α (TNF-α). This delivery led to downregulation of MMP-3 expression and upregulation of aggrecan (a key component of ECM) expression, shifting IVD cells toward a more anabolic state [136]. Recently, targeted CRISPR activation (CRISPRa) of a novel zinc-finger gene *ZNF865* in human stem cells significantly enhances cartilage ECM productivity; engineered cells accumulate 8.5-fold more proteoglycans and 88.6-fold more collagen II in tissue-engineered IVD [137]. In fibrotic models, Daliri *et al.* [138] used CRISPR base editing of the COL1A1 promoter in fibroblasts, creating a point mutation that reduced COL1A1 transcription and collagen I secretion. These insights are particularly valuable for addressing SNPs such as SP1, which are known to dysregulate COL1A1 expression. Given its established role in altering collagen type I homeostasis, CRISPR-based approaches could offer a promising avenue to normalize COL1A1 transcription in individuals carrying the SP1 variant. By selectively modulating gene expression at the promoter level, these technologies may help restore ECM balance and reduce susceptibility to AF degeneration; however, this therapeutic potential warrants further investigation.

### 6.2 RNA-based Approaches

RNA-based approaches are an advanced, rapidly evolving class of therapies that function by regulating gene expression at the RNA level, without altering the underlying DNA sequence. In the context of DDD, these therapies aim to either inhibit pathological genes (such as matrix-degrading enzymes) or increase the expression of protective [139]. RNA-based approaches demonstrated the potential to restore ECM homeostasis by downregulating catabolic signals or upregulating anabolic proteins such as collagen II and aggrecan. The multifunctionality of RNA-based therapies, along with improvements in delivery systems such as hydrogels, positions them as promising non-surgical strategies to slow or reverse IVD degeneration [140, 141].

In the context of COL1A1, Schuppan *et al.* [142] introduced lipid-nanoparticle (LNP)-encapsulated siRNA against Col1a1 into mice with hepatic fibrosis. This method achieved ~90% knockdown of procollagen I mRNA and a 40–60% reduction in collagen deposition in fibrotic livers. The translation of this method into chondrocyte-like cells *in vitro* derived from degenerative IVD, characterized by increased COL1A1 expression, is possible but requires further research. Additionally, a study using an OA rat model developed an injectable LNP-siRNA system to suppress Indian hedgehog (Ihh). As a result, collagen type II expression increased, and the hallmarks of cartilage degeneration decreased [143]. Similarly, LNA antisense against miR-449a in rodent OA upregulated collagen II and aggrecan, promoting cartilage regeneration [144]. Moreover, ADAMTS-5 siRNA has shown potential for IVD regeneration; injection into a rabbit model with DDD inhibited ECM degradation and improved the histological grade of the NP tissue. Additionally, osteogenic protein-1 (OP-1) demonstrated an anticatabolic effect by blocking TNF-α-induced upregulation of both ADAMTS-4 and ADAMTS-5, thereby reversing TNF-α-mediated degradation of aggrecan and collagen type II in human IVD tissue *in vitro*. These findings collectively support RNA-based interventions as promising tools for modulating degenerative pathways and preserving ECM in IVD tissues [145].

## 7 FUTURE PERSPECTIVES

Genetic research has the potential to greatly improve our understanding of musculoskeletal diseases, including DDD. Further studies will involve thorough genetic profiling of various populations to identify the risk and protective alleles. Gene-environment interactions, especially those driven by lifestyle factors, would also be an integral part of a comprehensive understanding of DDD pathogenesis.

The combination of gene-editing technologies, such as CRISPR/Cas9, may potentially enable the *in vivo* correction of pathogenic mutations, whereas biomaterials selected through genetic insights may allow the creation of bioengineered tissues that mimic the properties of healthy IVDs. Finally, accurate translation to therapies will involve collaborative clinical trials among geneticists, clinicians, and bioengineers. The expansion of genetic profiling across diverse populations is a key focus for future research. This effort will enhance our understanding of the full spectrum of genetic variations that contribute to musculoskeletal disorders by identifying both risk alleles and protective variants specific to different populations. Detailed genetic mapping will elucidate the molecular epidemiology of these conditions and facilitate targeted interventions.

Another important avenue for exploration is the interplay between genetic predisposition and environmental factors, including lifestyle, diet, and physical activity. Understanding these interactions requires longitudinal studies and sophisticated analytical models that could provide a more comprehensive view of the mechanisms underlying these diseases. This holistic approach is crucial for developing effective prevention strategies that consider both genetic and environmental factors.

The application of personalized medicine for the treatment of musculoskeletal disorders represents a promising approach. By tailoring preventive and therapeutic interventions to individual genetic profiles, clinicians can optimize treatment efficacy and minimize adverse effects, heralding a new era of personalized health care. Furthermore, rapid advances in gene-editing technologies, particularly CRISPR/Cas9, offer the potential to correct pathological genetic variants *in vivo*. Research on gene therapy applications to strengthen collagen networks within the spine could lead to revolutionary treatments for DDD and related conditions.

Innovations in biomaterials and tissue engineering have shown significant potential. By integrating genetic insights into the development of biomaterials, researchers can create engineered tissues that replicate the genetic and structural properties of healthy IVD. Such developments could provide durable and effective treatments for patients with degenerative conditions.

To bring these genetic discoveries to clinical application, robust clinical trials are essential. These trials should test for new diagnostics, treatments, and preventive strategies derived from genetic research. Collaborative efforts that bring together geneticists, clinicians, and bioengineers will be crucial for effectively transitioning from bench to bedside.

Finally, as genetic technology advances, there is a pressing need to address educational and ethical considerations. Healthcare providers and patients must be informed of the potential limitations of genetic therapies and interventions. Ensuring ethical deployment and equitable access to these innovations will be critical as we move forward in genetic research on musculoskeletal diseases.

## CONCLUSION

This review highlights the essential role of collagen types I, II, IX, and XI in maintaining the structural integrity and biomechanical properties of the IVD and related connective tissues. Genetic variations within their corresponding genes—*COL1A1*, *COL2A1*, *COL9A2*, *COL9A3*, *COL11A1*, and *COL11A2*—have been linked to DDD and related musculoskeletal disorders. Among these, the SP1 polymorphism in *COL1A1* is more common and more strongly associated with DDD in Caucasian populations than in Asian populations, suggesting population-specific genetic susceptibility. However, its functional consequences remain unclear and warrant further *in vitro* investigation, particularly in fibroblasts derived from IVD tissue.

In contrast, evidence for other COL1A1 variants is still limited. The intronic SNP rs2075555 has been investigated in only two studies to date, both in Northern European (Caucasian) cohorts. In the Finnish study, the association was reported in a single radiological feature (disc signal intensity), whereas the Norwegian study evaluated DDD in a broader clinical and radiographic context. Moreover, the restriction of both studies to Caucasian populations underscores the need to examine rs2075555 in non-Caucasian ethnicities to determine whether its effects are population-specific or more generalizable.

Similarly, the available data for rs909102 are based on only two studies, and one of these did not include a control group, substantially limiting the ability to infer a robust association with DDD. Overall, while COL1A1 remains a strong biological candidate given its central role in AF and bone matrix integrity, the current genetic evidence beyond SP1 remains fragmented. Larger, well-controlled, and ethnically diverse studies, combined with functional analyses, are required to clarify which COL1A1 variants truly contribute to DDD susceptibility.

For COL2A1, several polymorphisms have been investigated in relation to DDD, but the strength of evidence varies across loci. From a biological standpoint, COL2A1 is a key structural component of the NP, yet functional studies directly assessing how specific COL2A1 variants alter ECM biomechanics remain limited. Addressing this gap through *in vitro* and *ex vivo* functional analyses is essential to move beyond purely associative findings. The intronic variant rs2276454 has shown relatively consistent associations with DDD in Korean and Han Chinese populations, supporting its role as a promising susceptibility marker in East Asians. A meta-analysis of existing studies could provide a more precise estimate of effect size and heterogeneity, while additional studies in other populations are required to determine whether this association extends beyond East Asian groups. The evidence for other COL2A1 polymorphisms is more preliminary. The rs1793953 variant has been reported to exert a potentially protective effect in only one study, necessitating independent replication in larger, ethnically diverse cohorts before any firm conclusions can be drawn. Although rs1793937 and rs2070739 have so far yielded null results, these findings are based on a limited number of investigations. Overall, while COL2A1 remains a biologically plausible candidate gene for DDD, the current genetic evidence still requires consolidation through meta-analyses, replication across populations, and mechanistic functional work.

Variants in COL9A2 and COL9A3 further illustrate the genetic and methodological complexity underlying DDD. Trp2, although more frequent in Asian populations, has paradoxically shown stronger disease associations in Caucasian cohorts. This discrepancy likely reflects a combination of methodological differences, variation in phenotype definitions, and limited sample sizes rather than a simple frequency–effect relationship. A critical issue is the frequent misinterpretation of Trp2 and Arg2 in several studies: because these substitutions occur at the same codon, some reports have referred to Arg2 carriers as Trp2 carriers or failed to clearly distinguish between them. To avoid such misclassification, future work should prioritize direct DNA sequencing over RFLP-based methods. The same concern applies to COL9A3 Trp3 and Arg3, where codon-level overlap can lead to ambiguity if genotyping strategies are not sufficiently specific. Trp3 variant appears to exert context-dependent effects, with associations reported predominantly in males or in obese individuals, suggesting that gene–sex and gene–environment interactions may modulate its impact on disc degeneration. Beyond Trp3 and Arg3, other COL9A3 variants, such as rs6122316 and the Arg-384 substitution, have been associated with LDH in only two separate studies. While these findings are intriguing, they currently rest on limited evidence and require independent replication in larger and ethnically diverse cohorts. Moreover, both studies focused specifically on LDH, underscoring the need to explore the effects of these variants across a broader spectrum of DDD phenotypes.

Polymorphisms in *COL11A1* and *COL11A2*, including rs1676486 and IVS6–4A>T, have also been associated with disc herniation and spinal stenosis, though these associations vary depending on spinal region, age, and study design. For COL11A1 rs1676486, both case–control studies and meta-analytic data suggest that the Ser allele confers only a modest, population-dependent risk, with some cohorts showing stronger effects in the cervical spine than in the lumbar region, whereas rs1337185 has yielded inconsistent results across spinal levels and study populations. Similarly, for COL11A2, variants such as rs9277935 and IVS6–4A>T show discordant findings between Chinese and Finnish cohorts, and additional SNPs (*e.g*., rs2071025, rs2076311) display either population-specific associations or no apparent effect.

These findings illustrate that genetic susceptibility to DDD is multifactorial and shaped by interactions with environmental, lifestyle, and demographic factors. As such, a more personalized approach to prevention and treatment that accounts for individual genetic background holds significant promise.

To advance our understanding of the genetic underpinnings of DDD, future research must prioritize large-scale, ethnically diverse studies with standardized diagnostic frameworks, age- and sex-stratified analyses, and functional validation of candidate SNPs. For missense variants, a predictive model is warranted to assess their structural and functional impact. Recent advances in biotechnology, particularly CRISPR/Cas9, offer opportunities not only to better understand collagen-related genetic mechanisms but also to explore targeted interventions. Integrating such technologies with genetic insights brings us closer to predictive, preventive, and personalized medicine, with the potential to improve outcomes for patients affected by DDD and related conditions.

## Figures and Tables

**Fig. (1):**
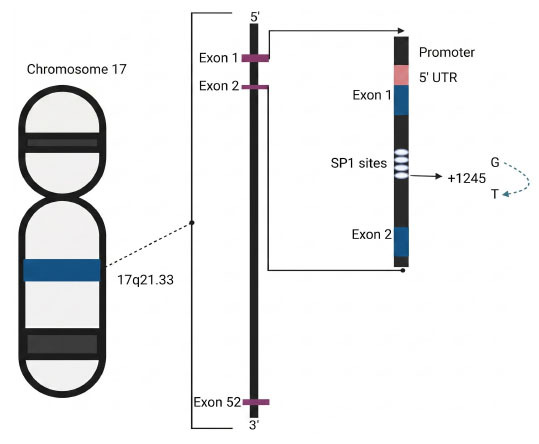
Genomic location of the +1245G>T SP1-binding site polymorphism in COL1A1. This SNP disrupts SP1 binding and alters the relative expression of COL1A1 and COL1A2, thereby disrupting the balance of type I collagen chains.

**Fig. (2):**
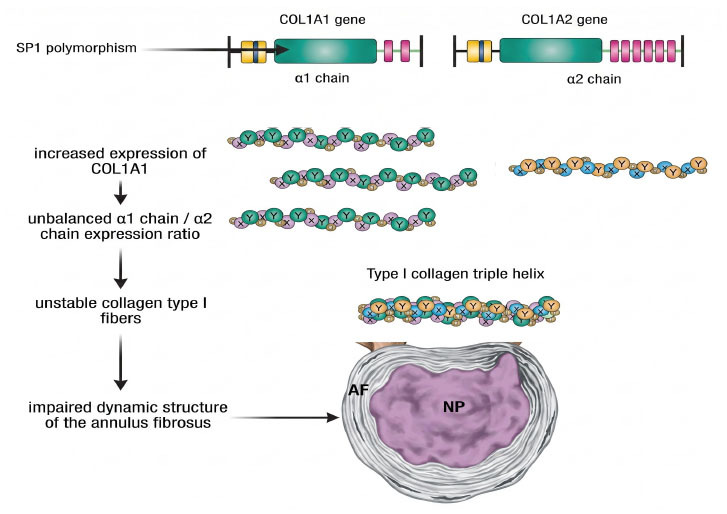
Mechanism by which SP1 polymorphism destabilizes collagen type I fibrils. An altered COL1A1/COL1A2 expression ratio compromises AF structural integrity.

**Fig. (3):**
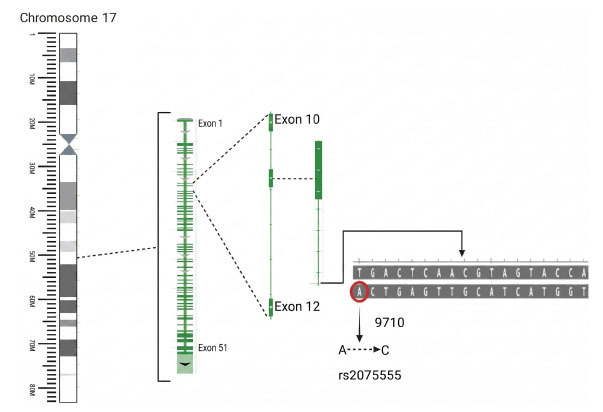
Position of the rs2075555 SNP in the 3′ region of the COL1A1 gene. Located at nucleotide position 9710, adjacent to exon 11, and involved an adenine (A) to cytosine (C) substitution.

**Fig. (4):**
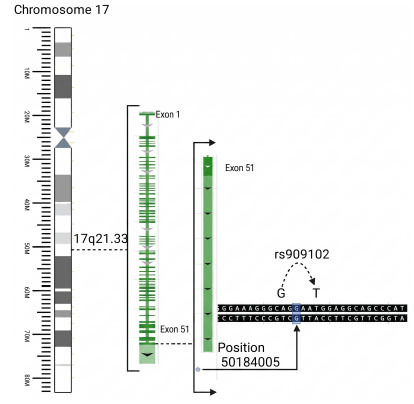
Genomic location of COL1A1 SNP rs909102. This variant is positioned at nucleotide 50,184,005, near exon 51, and involves a guanine (G) to thymine (T) substitution.

**Fig. (5):**
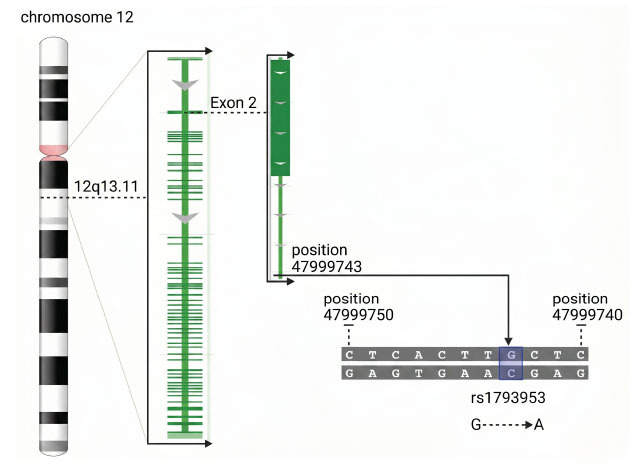
Genomic location of SNP rs1793953 in the COL2A1 gene. This variant is located near exon two at nucleotide position 47,999,750 and involves a guanine (G) to adenine (A) substitution.

**Fig. (6):**
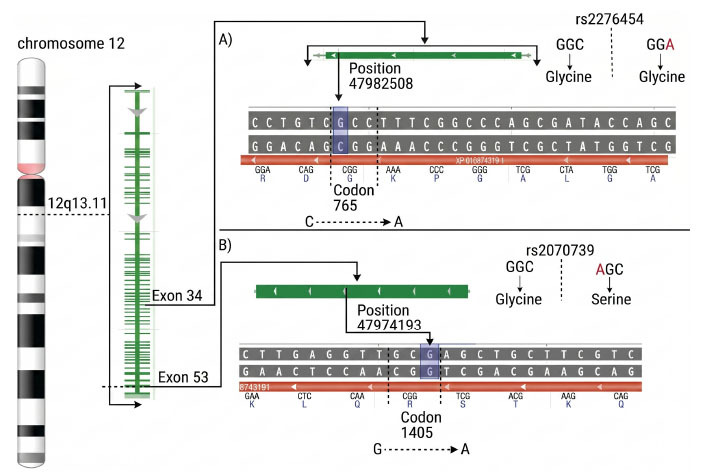
Genomic positions and nucleotide variations of SNPs in the COL2A1 gene. (**A**) SNP rs2276454 is located in exon 34 and involves a codon change at position 765 from GGA to GGC, both of which encode for glycine. (**B**) SNP rs2070739 is located in exon 53 and changes codon 1405 from GGC (glycine) to AGC (serine), resulting in a missense mutation.

**Fig. (7):**
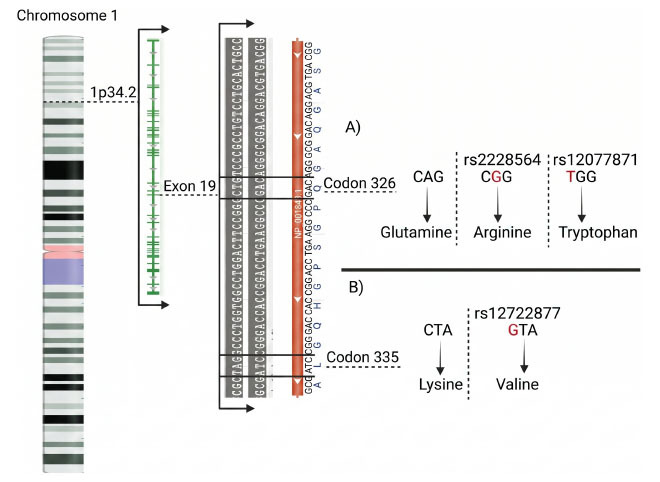
Detailed Codon Changes and Associated SNPs in Exon 19: (A) Codon 326 showing changes from CAG (Glutamine) to CGG (Arginine) and TGG (Tryptophan) for SNPs rs2228564 and rs12077871; (B) Codon 335 showing a change from CTA (Lysine) to GTA (Valine) for SNP.

**Fig. (8):**
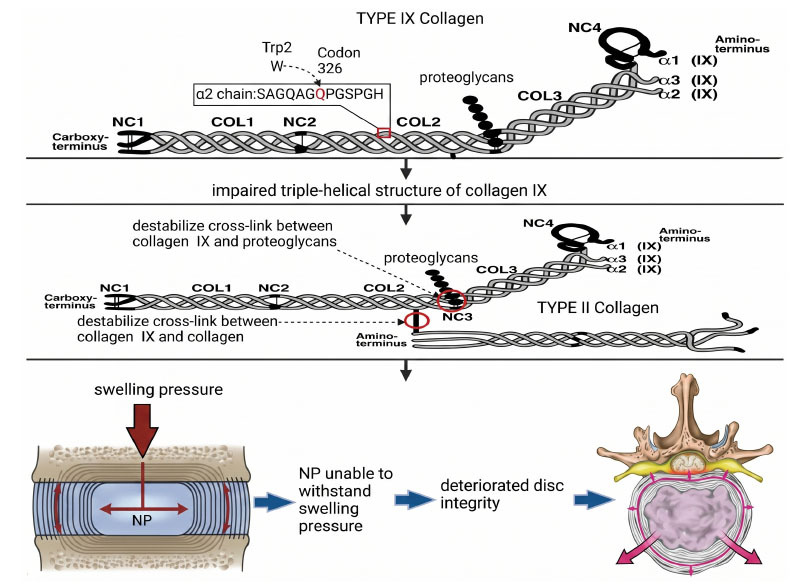
Pathophysiological impact of Trp2 substitution on NP integrity. A missense mutation at codon 326 of COL9A2 replaces glutamine with tryptophan (W), impairing the triple helical structure of collagen IX and weakening its cross-links with collagen II and proteoglycans. This disruption compromises ECM cohesion and reduces the NP’s ability to withstand swelling pressure, ultimately leading to disc degeneration. (W = tryptophan, Q = glutamine).

**Fig. (9):**
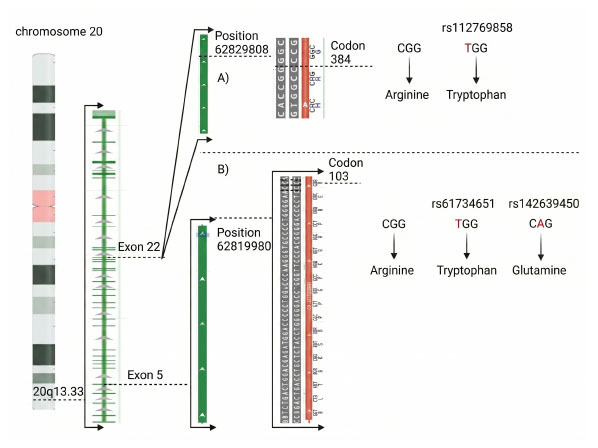
Detailed Codon Changes and Associated SNPs in COL9A3: A) Codon 384 showing changes from CGG (Arginine) to TGG (Tryptophan) for SNP rs112769858; B) Codon 103 showing changes from CGG (Arginine) to TGG (Tryptophan) for SNP rs61734651 and from CGG (Arginine) to CAG (Glutamine) for SNP rs142639450.

**Fig. (10):**
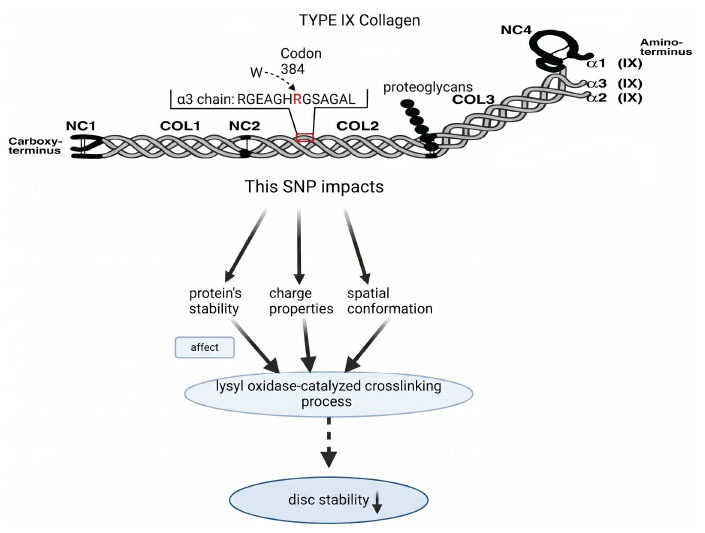
Impact of Arg384Trp polymorphism in COL9A3 on protein function and disc integrity. A substitution at codon 384 in the α3 chain of type IX collagen changes arginine to tryptophan, potentially altering the protein stability, charge distribution, and spatial conformation. These changes may impair lysyl oxidase-mediated crosslinking with ECM components, ultimately reducing the IVD stability. (R = arginine and W = tryptophan).

**Table 1:** Summary of COL1A1 SNPs associated with degenerative DDD in different populations. Genotype and allele frequencies, along with reported associations with DDD, are shown for key variants, including SP1, rs2075555, and rs909102.

Population	Sample Size (cases/control)	Gender	Genotypes/alleles	Cases (%)	Control (%)	*P*-value	Relation with DDD	Study ID
SP1 (rs1800012) (G/T)								
**Han Chinese**	375/118	Both male and female	GG	82 (69.5)	264(70.4)	0.009	Present	[45]
			GT	26 (22)	102(27.2)			
			TT	10(8.5)	9(2.4)			
			G	190(80.5)	630(84)	0.211	Absent	
			T	46(19.5)	46(16)			
**Greek**	24/12	Only describe young soldiers	GG	6(25)	4(33.3)	0.001	Present	[46]
			GT	10(41.7)	8(66.7)			
			TT	8(33.3)	0(0)			
			G	22(45.8)	16(66.6)	0.1336*	Absent	
			T	26(54.1)	8(33.3)			
**Indian**	50/50	Both	GG	38(76)	39(78)	0.6744	Absent	[35]
			GT	10(20)	10(20)			
			TT	2(4)	1(2)			
			G	86(86)	88(88)	0.8339*		
			T	14(14)	12(12)			
**Turkish**	75/25	Only male	GG	60(60)	N/M	0.013	Present	[47]
			GT	32(32)				
			TT	8(8)				
			G	152(76)	N/M			
			T	48(24)				
**Netherlands**	493	Both	GG	346(70.18)	No Control	N/M	Present	[36]
			GT	130(26.36)				
			TT	17(3.46)				
			G	822(83.3)				
			T	164(16.7)				
**Finnish**	588 cases	Only male	TT	N/M	No Control	N/M	Absent	[29]
			GT					
			TT					
			G	1035(88)				
			T	141(12)				
rs2075555 (A/C)								
**Finnish**	588 cases	Only male	AA	N/M	No Control	N/M	Present (associated with disc signal intensity)	[29]
			AC					
			CC					
			A	1123(87)				
			C	153(13)				
**Norwegian**	146/188	Both	AA	N/M		N/M	Absent	[50]
			AC					
			CC					
			A	37(12.7)	51(13.6)	0.4*		
			C	255(87.3)	325(86.4)			
rs909102 (T/C)								
**Iranian**	96/94	Both	TT	64 (66.7)	57 (60.6)	0.6	Absent	[12]
			TC	29 (30.2)	35 (37.2)	0.6		
			CC	3 (3.1)	2 (2.1)	1		
			T	157 81.8)	149 (79.3)	0.35		
			C	35 (18.2)	39 (20.7)	0.45		
**Finland**	588 cases	Only male	TT	N/M	No Control	N/M	Absent	[29]
			TC					
			CC					
			T	1011 (86)				
			C	165 (14)				

**Note:** (*) *p*-value was calculated using GraphPad InStat Version: 3.10.

N/M Not mentioned.

**Table 2:** Overview of COL2A1 SNPs and their associations with DDD. This table presents nucleotide substitutions, genotype and allele frequencies, and *p*-values for key COL2A1 variants, including rs1793953, rs2276454, rs1793937, and rs2070739, across multiple studies.

SNP	Nucleotide Substitution	Sample Size (cases/controls)	Genotypes/alleles	Cases (%)	Control (%)	*P*-value	Study ID
**rs1793953**	G/A	295 /324	GG	101(34.2)	97(30.0)	-	[56]
			GA	152(51.5)	148(45.7)		
			AA	42(14.3)	79(24.3)		
			G	354(60.0)	342(52.8)	0.010	
			A	236(40)	306(47.2)		
**rs2276454**	T/C	295 /324	TT	40(13.6)	121(37.3)	-	[56]
			TC	191(64.3)	150(46.3)		
			CC	64(21.7)	53(16.4)		
			T	271(45.9)	392(60.5)	<0.001	
			C	319(54.1)	256(39.5)		
**rs2276454**	T/C	51/235	TT	7(13.7)	87(37.0)	0.008† 0.001‡	[54]
			TC	33(64.7)	108 (46.0)		
			CC	11(21.6)	40(17.0)		
			T	47(46.1)	282(60.0)		
			C	55(53.9)	188(40.0)		
**rs2276454**	T/C	354/310	TT	50(14.1)	115(37.1)	<0.001	[57]
			TC	226(63.8)	142(45.8)		
			CC	78(22.1)	53(17.1)		
			T	326(46.1)	370(60)	<0.001	
			C	382(53.9)	248(40)		
**rs1793937**	C/G	295/324	CC	71(24.1)	86(26.5)	-	[56]
			CG	147(49.8)	158(48.8)		
			GG	77(26.1)	80(24.7)		
			C	289(49)	330(50.9)	0.495	
			G	301(51)	318(49.1)		
**rs2070739**	C/T	354 /310	CC	70(19.7)	53(17.1)	0.393	[57]
			CT	176(49.7)	148(47.7)		
			TT	108(30.5)	109(35.2)		
			T	316(44.6)	254(40.9)	0.178	
			C	392(55.4)	366(59.1)		

**Note:** (†) *p*-value for Codominant model.

(‡) *p*-value for Dominant model.

**Table 3:** Association of Trp2 (rs12077871) and Arg2 (rs2228564) alleles of COL9A2 with DDD. Codon 326 variants (Trp2, Arg2, Gln2, stop) are shown with their allele frequencies, *p*-values, and association types across populations.

-	**Trp2 (rs12077871) (996C/T) and Arg2 (rs2228564) (997A/G) of COL9A2 Codon Number 326 (TGG (Trp2), CGG (Arg2), CAG (Gln2), TAG (Stop codon)**								
**Population**	**Sample Size (cases/controls)**	**Gender**	**Alleles Frequency**	**Cases (%)**	**Control (%)**	***P*-value**	**Relation with** **DDD**	**Type of Association**	**Study ID**
**Finnish**	157/ 174	Unknown	Gln2	72.6%ᵃ	73.8%ᵃ	0.01	Trp2 associated with DDD	N/M	[59]
			Trp2	1.9%ᵃ	0				
			Arg2	25.5%ᵃ	26.2%ᵃ				
**German**	250 cases	Both	Trp2	1.2% ᵇ	No control	N/M	Present	Trp2 is an age-dependent risk factor	[70]
			Gln2 or Arg2	98.8%ᵇ					
**Southern Chines**	514/290	Both	Trp2	19.9% ᵇ	N/M	N/M	Present	Trp2 is an age-dependent risk factor for DDD (age group 40 to 49 years)	[72]
								Trp2 is an age-dependent risk factor for annular tears (age group 30 to 39 years )	
			Gln2 or Arg2	80.09% ᵇ					
**North Indian**	100/100	Both	Trp2	21% ᵇ	10% ᵇ	0.03	Present	Trp2 is associated with severity	[73]
			Gln2 or Arg2	79%ᵇ	90%ᵇ				
**Japanese**	84 cases	Both	Trp2	21.4% ᵇ	No control	N/M	Present	Trp2 is associated with severity in the age group under 40	[80]
			Gln2 or Arg2	78.6% ᵇ					
**Japanese**	470 /658	Both	Trp2	11.59%ᵃ	12.53%ᵃ	0.500	Absent	-	[74]
			Arg2	18.72%ᵃ	16.28%ᵃ	0.443			
			Gln2	69.68%ᵃ	71.17%ᵃ				
**Iranian**	96/94	Both	Trp2	22.8%ᵃ	22.9%ᵃ	1.00	Absent	-	[12]
			Gln2 or Arg2	77.2%ᵃ	77.1%ᵃ				
**Turkish**	75/25	Only male	Trp2	1.7%ᵃ	0	0.119	Absent	-	[47]
			Gln2 or Arg2	98.3%ᵃ	100%ᵃ				
**Iranian**	108/57	Both	Trp2	31.5% ᵇ	22.8%ᵇ	0.297	Absent	-	[82]
			Gln2 or Arg2	68.5%ᵇ	77.2%ᵇ				
**Greece**	105/103	Only male	Trp2	0	N/M	N/M	Absent	-	[75]
			Arg2	22%ᵃ					
			Gln2	78%ᵃ					
**German**	144 /144	Both	Trp2	0	0	-	-	-	[85]
			Arg2	14.2%ᵃ	10.06%ᵃ	0.1248	Absent		
			Gln2	85.8%ᵃ	89.93%ᵃ				
**Chinese**	215/230	Both	Trp2	-	-	-	-	Homozygous Arg2 increases the risk by 2.42-fold	[86]
			Arg2	42.09%ᵃ	36.30%ᵃ	0.0857*	Present		
			Gln2	57.09%ᵃ	63.69%ᵃ				
						-	-		
**Chinese Singaporean**	34/20	Only male	Trp2	7.35%ᵃ	7.5%ᵃ	0.596 in comparison with Arg2 or Gln2	Absent	-	[83]
			Arg2	47.05%ᵃ	52.5%ᵃ	0.373	-	-	
			Gln2	45.58%ᵃ	40%ᵃ				
**Indian**	100/ 100	Both	Trp2	36%ᵃ	8.5%ᵃ	< 0.0001*	Present	-	[81]
			Gln2 or Arg2	64%ᵃ	91.5%ᵃ				

**Note:** (ᵃ) indicates the allele frequency in the population.

(ᵇ) indicates the percentage of individuals who carry Trp2, Arg2 and/or Gln2 without knowing if they are homozygous or heterozygous.

(*) *P*-value is calculated using GraphPad InStat Version: 3.10.

N/M Not mentioned.

**Table 4:** Association of rs12722877 in the COL9A2 gene with DDD. Genotype and allele frequencies, *p*-values, and types of association are presented across study populations.

-	rs12722877 ( 1003C/G) 335 Leu/Val								
Population	**Sample Size (cases/controls)**	**Gender**	**Genotypes/alleles**	**Cases (%)**	**Control (%)**	***P*-value**	**Relation with DDD**	**Type of Association**	**Study ID**
**Japanese**	470/658	Both male and female	CC	414 (88.27%)	576 (88.08%)	0.955	Absent	-	[74]
			CG	54(11.51%)	76 (11.62)				
			GG	1 (0.21%)	2 (0.30%)				
			C	882 (94.02%)	1228 (93.88%)	0.886	Absent	Absent	
			G	56(5.9%)	(80) 6.11%				
**Chinese Singaporean**	34/20	Only male	CC	27 (79.41%)	10 (50%)	0.015	Present	Val allele is a protective allele (reduces the risk of DDD)	[83]
			CG	5 (14.7%)	10 (50%)				
			GG	2 (5.88%)	0				
			C	59 (86.76%)	30 (75%)	0.1895*	Absent		
			G	9 (13.23)	10 (25%)				
**Chinese**	280/268	Both male and female	CC	158 (56.4%)	120 (44.8%)	0.01	Present	-	[88]
			CG	98 (35%)	113 (42.2%)				
			GG	24 (8.6%)	35(11%)				
			C allele	414 (74 %)	353 (65.9%)	0.004	Present	-	
			G allele	146 (26%)	183 (34.1%)				
**Finland**	171/ 321	Unknown	CC	N/M				-	[87]
			CG						
			GG						
			C	290 (92.94%)	312 (93.41%)	0.8764*	Absent	-	
			G	22 (7.05%)	22 (6.58%)				

**Note:** (*) *P*-value calculated using GraphPad InStat Version: 3.10.

N/M Not mentioned.

**Table 5:** Association of Trp3 (rs61734651) and Arg2 (rs142639450) alleles of COL9A3 with DDD. Codon 103 variants (Trp3, Arg3, Gln3, stop) are shown with their allele frequencies, *p*-values, and association types across populations.

-	Trp3 (rs61734651) (307C/T) and Arg3 (rs142639450) (308A/G) of COL9A3 Codon Number 103 (TGG (Trp3), CGG (Arg3), CAG (Gln3), TAG (stop codon)								
Population	**Sample Size (cases/controls)**	**Gender**	**Alleles Frequency**	**Cases (%)**	**Control (%)**	***P*-value**	**Relation with** **DDD**	**Type of Association**	**Study ID**
**Finland**	171 / 321	Unknown	Gln3	0.29%ᵃ	0.311%ᵃ	0.000013	Trp3 associated with DDD	N/M	[87]
			Trp3	12.28%ᵃ	4.67%ᵃ				
			Arg3	87.42%ᵃ	95.01%ᵃ				
**Finland**	135 cases	only male	Trp3	17%ᵇ	No control	N/M	Present	Trp3 is associated with DDD in obese individuals	[95]
			Gln3 or Arg3	83%ᵇ					
**Finland**	135 cases	Only male	Trp3	10.38%ᵃ	7.27%ᵃ	0.5156*	Present	The Trp3 allele is associated with DDD in the absence of L-1β(C 3954 -T) SNP	[96]
			Gln3 or Arg3	89.61%ᵃ	92.72%ᵃ				
**Iranian**	108 /57	Both	Trp3	26.9% ᵇ	17.5%	0.247	Present	The Trp3 allele is associated with DDD in the male cohort	[82]
			Gln3 or Arg3	73.1%	82.5%				
**Indian**	100 / 100	Both male and female	Trp3	2.5%ᵃ	3.5%ᵃ	0.7710*	Absent	-	[81]
			Gln3 or Arg3	97.5%ᵃ	96.5%ᵃ				
**Finland**	292/246	Both male and female	Trp3	14.67%ᵇ	21.13%ᵇ	N/M	Absent	-	[22]
			Arg3 or Gln3	85.27%ᵇ	78.86%ᵇ				
**American**	107 cases	Both male and female	Trp3	3.27%ᵃ	No control	N/M	Present	Trp3 is associated with spinal stenosis with spondylolisthesis	[65]
			Gln3 or Arg3	96.72%ᵃ					
**Turkish**	75 /25	Only male	Trp3	3.3%ᵃ	0	0.044	Present	Trp3 is associated with severity	[47]
			Gln3 or Arg3	96.6%ᵃ	100%ᵃ				
**Danish**	66/154	Both	Trp3	6.8%ᵃ	10.4%ᵃ	0.2851*	Absent	-	[97]
			Gln3 or Trp3	93.1%ᵃ	89.6%ᵃ				
**Greece**	105/103	Only male	Trp3	4.3%ᵃ	N/M	N/M	Absent	-	[75]
			Arg3	93.8%ᵃ					
			Gln3	1%ᵃ					
**Chinese Singaporean**	34/20	Only male	Trp3	0	0	-	Absent	-	[83]
			Arg3	4.5%ᵃ	2.5%ᵃ	0.604			
			Gln3	95.5%ᵃ	97.5%ᵃ				

**Note:** (ᵃ) indicates the allele frequency in the population.

(ᵇ) indicates the percentage of individuals who carry Trp3, Arg3 and/or Gln3 without knowing if they are homozygous or heterozygous.

(*) *P-*value calculated using GraphPad InStat Version: 3.10.

N/M Not mentioned.

**Table 6:** Association of rs1676486 (COL11A1, 3968C/T; Pro1535Ser) with DDD. Genotype and allele frequencies, *p*-values, and reported associations with DDD across study populations.

rs1676486 (3968C/T) 1535 Pro/Ser									
Population	**Sample Size (cases/controls)**	**Gender**	**Genotypes/alleles**	**Cases (%)**	**Control (%)**	***P*-value**	**Relation with** **DDD**	**Type of Association**	**Study ID**
**Japanese**	188/179	Both male and female	CC	41.71%	54.54%	0.000099	Present	N/M	[103]
			CT	45.6%	38.31%				
			TT	12.67%	7.14%				
			C	65%	74%	0.000015	Present		
			T	35%	26%				
**Chinese**	647/532	Both male and female	CC	40.64%	51.12%	0.004	Present	The homozygous T allele is associated with high DDD severity	[110]
			CT	48.84%	41.54%				
			TT	10.51%	7.33%				
			C	65%	71.9%	< 0.001	Present		
			T	35%	28.1%				
**Japanese**	47/45	Only male	CC	37.8%	51.1%	0.035	Present	T allele is associated with cervical DDD	[111]
			CT	44.5%	46.8%				
			TT	17.8%	2.1%				
			C	60%	74.5%	0.036	Present		
			T	40%	25.5%				
**Japanese**	54/69	Only male	CC	42.6%	44.9%	0.3	Absent	T allele is not associated with lumbar DDD	[111]
			CT	51.9%	42%				
			TT	5.6%	13%				
			C	68.5%	65.9%	0.67	Absent		
			T	31.5%	34.1%				
**Japanese**	103/55	Both male and female	CC	65.9%	60%	0.2914*	Absent	T allele is associated with Limbus Vertebra not with DDD	[113]
			CT	20.5%	32.7				
			TT	13.6%	7.3%				
			C	76.1%	76.4%	1.0000*			
			T	23.9%	23.6%				

**Note:** (*) *P*-value calculated using GraphPad InStat Version: 3.10.

N/M Not mentioned.

**Table 7:** Association of rs1337185 (COL11A1) with DDD. Summary of population data, gender, study type, odds ratios, *p*-values, and reported associations with DDD across studies.

-	rs1337185							
Population	**Sample Size (cases/controls)**	**Gender**	**Study Type**	***P*-value**	**Odd Ratio**	**Relation with DDD**	**Type of Association**	**Study ID**
**Finnish Population**	588 cases	Only male	population-based twin study	0.015	N/M	Present	Associated with disc bulging	[19]
**Indian population**	DDD:222/110	Both male and female	cross-sectional, case-control study	0.6923	N/M	Absent	Not associated with the Pfirmann score	[117]
	Annular tear 137/195			0.5807	N/M	Absent	Not associated with a high-intensity zone	
	End-plate damage 198/134			0.7402	N/M	Absent	Not associated with total end-plate score	
**Japanese Population**	695	Both male and female	cross-sectional, case-control study	0.02433	1.55	Present	Associated with a severe Total Disc Degenerative Score	[27]
**Chinese Han population**	428/400	Both male and female	observational study	0.003	1.8	Present	Associated with a disc herniation	[116]
				0.004	1.92	Present	Associated with spinal stenosis and spondylolisthesis	

**Note:** N/M Not mentioned.

**Table 8:** COL11A2 variants associated with DDD across different populations. Includes various SNPs with details on the type of variation, population, gender, *p*-values, and reported associations with DDD.

Variation	Type of Variation	Sample Size (cases/controls)	Population	Gender	*P*-value	Relation with DDD	Type of Association	Study ID
**IVS6-4 A_T) rs1799907**	Intron Variant	150/61	Finnish Population	Only male	0.830	Absent	Not associated with DDD	[130]
		29	Finnish Population	Both male and female	0.0016	Present	Associated with Spinal stenosis	[129]
		195/187	Japanese Population	Unknown	0.0003	Present	Associated with OPLL	[128]
		66/154	Danish population	Male and female	0.1975*	Absent	Not associated with DDD	[97]
**A/G polymorphism**	Intron 9	135 cases	Finnish Population	Only male	N/M	Present	Associated with disc bulges	[96]
**rs2072915**	3 Prime UTR Variant	588 cases	Finnish Population	Only male	0.029	Present	Associated with disc signal intensity	[29]
**rs9277933**	Intron Variant				0.03	Present	Associated with disc signal intensity	
					0.018	Present	Associated with disc signal intensity	
**rs2076311**	Intron Variant							
**rs2071025**	Intron Variant	384/384	Chinese Han population	Both male and female	0.0002	Present	Associated with DDD	[126]
**rs986522**	Intron Variant				0.019		Associated with DDD only in the female cohort	
**rs17214944**	Intron Variant				0.042		Associated with DDD under the dominant model	
					0.024		Associated with DDD under the Log‐additive model	

**Note:** (*) *P*-value is calculated using GraphPad InStat Version: 3.10.

N/M: Not mentioned.

OPLL: ossification of the posterior longitudinal ligament.

## LIST OF ABBREVIATIONS

AF: Annulus Fibrosus
BMI: Body Mass Index
DDD: Degenerative Disc Disease
DLS: Degenerative Lumbar Scoliosis
ECM: Extracellular Matrix
Ihh: Indian Hedgehog
IVD: Intervertebral Disc
LBP: Low Back Pain
LDH: Lumbar Disc Herniation
LSS: Lumbar Spinal Stenosis
MMPs: Matrix Metalloproteinases
NP: Nucleus Pulposus
OP-1: Osteogenic Protein-1
OPLL: Ossification of the Posterior Longitudinal Ligament
RFLP: Restriction Fragment Length Polymorphism
SNPs: Single-nucleotide Polymorphisms
TDDS: Total Disc Degenerative Score
TNF-α: Tumor Necrosis Factor-α

## References

[1] Schol J., Sakai D. (2019). Cell therapy for intervertebral disc herniation and degenerative disc disease: Clinical trials.. Int. Orthop..

[2] Rahyussalim A.J., Zufar M.L.L., Kurniawati T. (2020). Significance of the association between disc degeneration changes on imaging and low back pain: A review article.. Asian Spine J..

[3] Biswas A., Garg B. (2024). Decoding the genetic threads of disc degeneration.. Indian Spine J..

[4] Chan W.C.W., Sze K.L., Samartzis D., Leung V.Y.L., Chan D. (2011). Structure and biology of the intervertebral disk in health and disease.. Orthop. Clin. North Am..

[5] Liebsch C., Wilke H.J. (2022). Even mild intervertebral disc degeneration reduces the flexibility of the thoracic spine: An experimental study on 95 human specimens.. Spine J..

[6] Dowdell J., Erwin M., Choma T., Vaccaro A., Iatridis J., Cho S.K. (2017). Intervertebral disk degeneration and repair.. Neurosurgery.

[7] Molladavoodi S., McMorran J., Gregory D. (2020). Mechanobiology of annulus fibrosus and nucleus pulposus cells in intervertebral discs.. Cell Tissue Res..

[8] Zhang S., Liu W., Chen S., Wang B., Wang P., Hu B., Lv X., Shao Z. (2022). Extracellular matrix in intervertebral disc: Basic and translational implications.. Cell Tissue Res..

[9] Martirosyan N.L., Patel A.A., Carotenuto A., Kalani M.Y.S., Belykh E., Walker C.T., Preul M.C., Theodore N. (2016). Genetic alterations in intervertebral disc disease.. Front. Surg..

[10] Mordechai H.S., Aharonov A., Sharon S.E., Bonshtein I., Simon C., Sivan S.S., Sharabi M. (2023). Toward a mechanically biocompatible intervertebral disc: Engineering of combined biomimetic annulus fibrosus and nucleus pulposus analogs.. J. Biomed. Mater. Res. A.

[11] Rajasekaran S., Soundararajan D.C.R., Nayagam S.M., Tangavel C., Raveendran M. (2023). K S, S.V.A.; Shetty, A.P.; Kanna, R.M. Novel biomarkers of health and degeneration in human intervertebral discs: In-depth proteomic analysis of collagen framework of fetal, healthy, scoliotic, degenerate, and herniated discs.. Asian Spine J..

[12] Hanaei S., Abdollahzade S., Sadr M., Fattahi E., Mirbolouk M.H., Khoshnevisan A., Rezaei N. (2021). Lack of association between COL1A1 and COL9A2 single nucleotide polymorphisms and intervertebral disc degeneration.. Br. J. Neurosurg..

[13] Murphy K., Lufkin T., Kraus P. (2023). Development and degeneration of the intervertebral disc—insights from across species.. Vet. Sci..

[14] Karchevskaya A.E., Poluektov Y.M., Korolishin V.A. (2023). Understanding intervertebral disc degeneration: Background factors and the role of initial injury.. Biomedicines.

[15] Liang H., Luo R., Li G., Zhang W., Song Y., Yang C. (2022). The proteolysis of ECM in intervertebral disc degeneration.. Int. J. Mol. Sci..

[16] Statzer C., Luthria K., Sharma A., Kann M.G., Ewald C.Y. (2023). The human extracellular matrix diseasome reveals genotype–phenotype associations with clinical implications for age-related diseases.. Biomedicines.

[17] Mern D.S., Walsen T., Beierfuß A., Thomé C. (2021). Animal models of regenerative medicine for biological treatment approaches of degenerative disc diseases.. Exp. Biol. Med..

[18] Taylor W., Erwin W.M. (2024). Intervertebral disc degeneration and regeneration: new molecular mechanisms and therapeutics: Obstacles and potential breakthrough technologies.. Cells.

[19] Feng Y., Egan B., Wang J. (2016). Genetic factors in intervertebral disc degeneration.. Genes Dis..

[20] Munir S., Rade M., Määttä J.H., Freidin M.B., Williams F.M.K. (2018). Intervertebral disc biology: Genetic basis of disc degeneration.. Curr. Mol. Biol. Rep..

[21] Sun Y., Lyu M., Lu Q., Cheung K., Leung V. (2022). Current perspectives on nucleus pulposus fibrosis in disc degeneration and repair.. Int. J. Mol. Sci..

[22] Kelempisioti A., Eskola P.J., Okuloff A., Karjalainen U., Takatalo J., Daavittila I., Niinimäki J., Sequeiros R.B., Tervonen O., Solovieva S., Kao P.Y.P., Song Y.Q., Cheung K.M.C., Chan D., Ala-Kokko L., Järvelin M.R., Karppinen J., Männikkö M. (2011). Genetic susceptibility of intervertebral disc degeneration among young Finnish adults.. BMC Med. Genet..

[23] Theodore N., Ahmed A.K., Fulton T., Mousses S., Yoo C., Goodwin C.R., Danielson J., Sciubba D.M., Giers M.B., Kalani M.Y.S. (2019). Genetic predisposition to symptomatic lumbar disk herniation in pediatric and young adult patients.. Spine.

[24] Wang Y., Battié M.C., Shapiro I.M., Risbud M.V. (2014). Epidemiology of lumbar disc degeneration.. The Intervertebral Disc: Molecular and Structural Studies of the Disc in Health. and Disease..

[25] Rigal J., Léglise A., Barnetche T., Cogniet A., Aunoble S., Le Huec J.C. (2017). Meta-analysis of the effects of genetic polymorphisms on intervertebral disc degeneration.. Eur. Spine J..

[26] Guo W., Li B.L., Zhao J.Y., Li X.M., Wang L.F. (2024). Causal associations between modifiable risk factors and intervertebral disc degeneration.. Spine J..

[27] Rajasekaran S., Kanna R.M., Senthil N., Raveendran M., Ranjani V., Cheung K.M.C., Chan D., Kao P.Y.P., Yee A., Shetty A.P. (2015). Genetic susceptibility of lumbar degenerative disc disease in young Indian adults.. Eur. Spine J..

[28] Zhang Y., Sun Z., Liu J., Guo X. (2008). Advances in susceptibility genetics of intervertebral degenerative disc disease.. Int. J. Biol. Sci..

[29] Videman T., Saarela J., Kaprio J., Näkki A., Levälahti E., Gill K., Peltonen L., Battié M.C. (2009). Associations of 25 structural, degradative, and inflammatory candidate genes with lumbar disc desiccation, bulging, and height narrowing.. Arthritis Rheum..

[30] Kanniyappan H., Chathurika Rathnayake R.A., Osamor J., Islam M., Wang R.R. (2025). The role of collagen and collagen I/III ratio in pathological conditions: Insights into molecular mechanisms and therapeutic approaches.. Front. Bioeng. Biotechnol..

[31] Henriksen K., Karsdal M.A. (2016). Type I Collagen.. Biochemistry of Collagens, Laminins and Elastin..

[32] Naomi R., Ridzuan P.M., Bahari H. (2021). Current insights into collagen type I.. Polymers.

[33] Yamauchi M., Sricholpech M. (2012). Lysine post-translational modifications of collagen.. Essays Biochem..

[34] Henriksen K., Leeming D.J., Christiansen C., Karsdal M.A. (2011). Use of bone turnover markers in clinical osteoporosis assessment in women: Current issues and future options.. Womens Health.

[35] Anjankar S.D., Poornima S., Raju S., Jaleel M.A., Bhiladvala D., Hasan Q. (2015). Degenerated intervertebral disc prolapse and its association of collagen I alpha 1 Spl gene polymorphism.. Indian J. Orthop..

[36] Pluijm S.M.F., van Essen H.W., Bravenboer N., Uitterlinden A.G., Smit J.H., Pols H A P., Lips P. (2004). Collagen type I α1 Sp1 polymorphism, osteoporosis, and intervertebral disc degeneration in older men and women.. Ann. Rheum. Dis..

[37] Lu Y., Zhang S., Wang Y., Ren X., Han J. (2019). Molecular mechanisms and clinical manifestations of rare genetic disorders associated with type I collagen.. Intractable Rare Dis. Res..

[38] Xie P., Liu B., Zhang L., Chen R., Yang B., Dong J., Rong L. (2015). Association of COL1A1 polymorphisms with osteoporosis: A meta-analysis of clinical studies.. Int. J. Clin. Exp. Med..

[39] Kurt-Sirin O., Yilmaz-Aydogan H., Uyar M., Seyhan M.F., Isbir T., Can A. (2014). Combined effects of collagen type I alpha1 (COL1A1) Sp1 polymorphism and osteoporosis risk factors on bone mineral density in Turkish postmenopausal women.. Gene.

[40] Leźnicka K., Żyżniewska-Banaszak E., Gębska M., Machoy-Mokrzyńska A., Krajewska-Pędzik A., Maciejewska-Skrendo A., Leońska-Duniec A. (2021). Interactions between gene variants within the COL1A1 and COL5A1 genes and musculoskeletal injuries in physically active caucasian.. Genes.

[41] Pavlovic S., Ugrin M., Micic S., Gasic V., Dimitrijevic J., Barteczko U. (2018). Using genetics for enhancement (Liberal Eugenics).. Clinical Ethics. At the Crossroads of Genetic and Reproductive Technologies..

[42] Cichocka E., Górczyńska-Kosiorz S.B., Niemiec P., Trautsolt W., Gumprecht J. (2025). The impact of selected COL1A1 and COL1A2 gene polymorphisms on bone mineral density and the risk of metabolic diseases in postmenopausal women.. Int. J. Mol. Sci..

[43] Millington-Ward S., McMahon H.P., Farrar G.J. (2005). Emerging therapeutic approaches for osteogenesis imperfecta.. Trends Mol. Med..

[44] Lyu F-J., Cui H., Pan H., Mc Cheung K., Cao X., Iatridis J.C., Zheng Z. (2021). Painful intervertebral disc degeneration and inflammation: From laboratory evidence to clinical interventions.. Bone Res..

[45] Lin P., Fu Q. (2014). Association of collagen type I alpha1 Sp1 polymorphism with intervertebral disc degeneration in Han elderly people.. Sichuan Da Xue Xue Bao Yi Xue Ban.

[46] Tilkeridis C., Bei T., Garantziotis S., Stratakis C.A. (2005). Association of a COL1A1 polymorphism with lumbar disc disease in young military recruits: Table 1.. J. Med. Genet..

[47] Toktaş Z.O., Ekşi M.Ş., Yılmaz B., Demir M.K., Özgen S., Kılıç T., Konya D. (2015). Association of collagen I, IX and vitamin D receptor gene polymorphisms with radiological severity of intervertebral disc degeneration in Southern European Ancestor.. Eur. Spine J..

[48] Zhong B., Huang D., Ma K., Deng X., Shi D., Wu F., Shao Z. (2017). Association of COL1A1 rs1800012 polymorphism with musculoskeletal degenerative diseases: A meta-analysis.. Oncotarget.

[49] Farias-Cisneros E., Hidalgo-Bravo A., Miranda-Duarte A., Casas-Ávila L., Rozental T.D., Velázquez-Cruz R., Valdés-Flores M. (2020). COL1A1, CCDC170, and ESR1 single nucleotide polymorphisms associated with distal radius fracture in postmenopausal Mexican women.. Climacteric.

[50] Omair A., Lie B.A., Reikeras O., Brox J.I. (2012). An association study of interleukin 18 receptor genes (IL18R1 and IL18RAP) in lumbar disc degeneration.. Open Orthop. J..

[51] Sahlman J., Inkinen R., Hirvonen T., Lammi M.J., Lammi P.E., Nieminen J., Lapveteläinen T., Prockop D.J., Arita M., Li S.W., Hyttinen M.M., Helminen H.J., Puustjärvi K. (2001). Premature vertebral endplate ossification and mild disc degeneration in mice after inactivation of one allele belonging to the Col2a1 gene for Type II collagen.. Spine.

[52] Gudmann N.S., Karsdal M.A. (2016). Type II collagen.. Biochemistry of Collagens, Laminins and Elastin..

[53] Li X., Yang S., Qin L., Yang S., Type I.I. (2021). Type II collagen-positive embryonic progenitors are the major contributors to spine and intervertebral disc development and repair.. Stem Cells Transl. Med..

[54] Hwang D.W., Kim K.T., Lee S.H., Kim J.Y., Kim D.H. (2014). Association of COL2A1 gene polymorphism with degenerative lumbar scoliosis.. Clin. Orthop. Surg..

[55] Barat-Houari M., Sarrabay G., Gatinois V., Fabre A., Dumont B., Genevieve D., Touitou I. (2016). Mutation update for COL2A1 gene variants associated with type II collagenopathies.. Hum. Mutat..

[56] Deng Y., Tan X.T., Wu Q., Wang X. (2017). Correlations between COL2A and Aggrecan genetic polymorphisms and the risk and clinicopathological features of intervertebral disc degeneration in a Chinese han population: A case–control study.. Genet. Test. Mol. Biomarkers.

[57] Li Y-Z., Li J., Zhang J., Lin Q. (2016). Association of COL2A and aggrecan polymorphisms with the susceptibility of intervertebral disc degeneration.. Int. J. Clin. Exp. Med..

[58] Murtagh C.F., Brownlee T.E., Rienzi E., Roquero S., Moreno S., Huertas G., Lugioratto G., Baumert P., Turner D.C., Lee D., Dickinson P., Lyon K.A., Sheikhsaraf B., Biyik B., O’Boyle A., Morgans R., Massey A., Drust B., Erskine R.M. (2020). The genetic profile of elite youth soccer players and its association with power and speed depends on maturity status.. PLoS One.

[59] Annunen S., Paassilta P., Lohiniva J., Perälä M., Pihlajamaa T., Karppinen J., Tervonen O., Kröger H., Lähde S., Vanharanta H., Ryhänen L., Göring H.H.H., Ott J., Prockop D.J., Ala-Kokko L. (1999). An allele of COL9A2 associated with intervertebral disc disease.. Science.

[60] Diab M., Wu J.J., Eyre D.R. (1996). Collagen type IX from human cartilage: A structural profile of intermolecular cross-linking sites.. Biochem. J..

[61] Shahrajabian M.H., Sun W. (2024). Mechanism of action of collagen and epidermal growth factor: A review on theory and research methods.. Mini Rev. Med. Chem..

[62] Teles Filho R.V., Abe G.M., Daher M.T. (2020). A influência genética na degeneração discal – Revisão sistemática da literatura.. Rev. Bras. Ortop..

[63] Kumar A., Kumar N., Pathak Z., Kumar H. (2022). Extra cellular matrix remodeling: An adjunctive target for spinal cord injury and intervertebral disc degeneration.. Neurospine.

[64] He Y., Karsdal M.A. (2016). Type IX Collagen.. Biochemistry of Collagens, Laminins and Elastin..

[65] Matsui Y., Mirza S.K., Wu J.J., Carter B., Bellabarba C., Shaffrey C.I., Chapman J.R., Eyre D.R. (2004). The association of lumbar spondylolisthesis with collagen IX tryptophan alleles.. J. Bone Joint Surg. Br..

[66] Zhang Z., Zhang J., Ding L., Teng X. (2014). Meta-analysis of the association between COL9A2 genetic polymorphisms and lumbar disc disease susceptibility.. Spine.

[67] Pihlajamaa T., Vuoristo M.M., Annunen S., Perälä M., Prockop D.J., Ala-Kokko L. (1998). Human COL9A1 and COL9A2 genes. Two genes of 90 and 15 kb code for similar polypeptides of the same collagen molecule.. Matrix Biol..

[68] Xu H., Dong R., Zeng Q., Fang L., Ge Q., Xia C., Zhang P., Lv S., Zou Z., Wang P., Li J., Ruan H., Hu S., Wu C., Jin H., Tong P. (2022). Col9a2 gene deletion accelerates the degeneration of intervertebral discs.. Exp. Ther. Med..

[69] Leite Pereira C., Grad S., Gonçalves R.M. (2023). Biomarkers for intervertebral disc and associated back pain: From diagnosis to disease prognosis and personalized treatment.. JOR Spine.

[70] Wrocklage C., Wassmann H., Paulus W. (2000). COL9A2 allelotypes in intervertebral disc disease.. Biochem. Biophys. Res. Commun..

[71] Zhu Y., Wu J.J., Weis M.A., Mirza S.K., Eyre D.R., Type I.X. (2011). Type IX collagen neo-deposition in degenerative discs of surgical patients whether genotyped plus or minus for COL9 risk alleles.. Spine.

[72] Jim J.J.T., Noponen-Hietala N., Cheung K.M.C., Ott J., Karppinen J., Sahraravand A., Luk K.D.K., Yip S.P., Sham P.C., Song Y.Q., Leong J.C.Y., Cheah K.S.E., Ala-Kokko L., Chan D. (2005). The TRP2 allele of COL9A2 is an age-dependent risk factor for the development and severity of intervertebral disc degeneration.. Spine.

[73] Ekramuddin E., Tewari V., Kumar A., Das M. (2023). Relation between TRP2 allele in COL9A2 gene and lumbar disc degeneration in a subset of north Indian population.. Al Ameen J. Med. Sci..

[74] Seki S., Kawaguchi Y., Mori M., Mio F., Chiba K., Mikami Y., Tsunoda T., Kubo T., Toyama Y., Kimura T., Ikegawa S. (2006). Association study of COL9A2 with lumbar disc disease in the Japanese population.. J. Hum. Genet..

[75] Kales S.N., Linos A., Chatzis C., Sai Y., Halla M., Nasioulas G., Christiani D.C. (2004). The role of collagen IX tryptophan polymorphisms in symptomatic intervertebral disc disease in Southern European patients.. Spine.

[76] Aladin D.M.K., Cheung K.M.C., Chan D., Yee A.F.Y., Jim J.J.T., Luk K.D.K., Lu W.W. (2007). Expression of the Trp2 allele of COL9A2 is associated with alterations in the mechanical properties of human intervertebral discs.. Spine.

[77] Mwale F., Billinghurst C., Wu W., Alini M., Webber C., Reiner A., Ionescu M., Poole J., Poole A.R. (2000). Selective assembly and remodelling of collagens II and IX associated with expression of the chondrocyte hypertrophic phenotype.. Dev. Dyn. Off. Publ. Am. Assoc. Anat..

[78] A. Banaszkiewicz, Banaszkiewicz P.A., Kader D.F. (2019). P. Structure and function of connective tissue. In postgraduate orthopaedics: Viva guide for the FRCS (Tr & Orth) examination..

[79] Khan A.N., Jacobsen H.E., Khan J., Filippi C.G., Levine M., Lehman R.A.J., Riew K.D., Lenke L.G., Chahine N.O. (2017). Inflammatory biomarkers of low back pain and disc degeneration: A review.. Ann. N. Y. Acad. Sci..

[80] Higashino K., Matsui Y., Yagi S., Takata Y., Goto T., Sakai T., Katoh S., Yasui N. (2007). The alpha2 type IX collagen tryptophan polymorphism is associated with the severity of disc degeneration in younger patients with herniated nucleus pulposus of the lumbar spine.. Int. Orthop..

[81] Rathod T.N., Chandanwale A.S., Gujrathi S., Patil V., Chavan S.A., Shah M.N. (2012). Association between single nucleotide polymorphism in collagen IX and intervertebral disc disease in the Indian population.. Indian J. Orthop..

[82] Bagheri M.H., Honarpisheh A.P., Yavarian M., Alavi Z., Siegelman J., Valtchinov V.I. (2016). MRI phenotyping of COL9A2/Trp2 and COL9A3/Trp3 alleles in lumbar disc disease.. Spine.

[83] Lim E.C.W., Wong W.P., Ng G.B.P., Chan L.L., Tan S.B., Tow P.B., Zhao Y. (2011). Distribution of COL9A2 and COL9A3 gene polymorphism in male Chinese Singaporean – A pilot observational study.. Int. J. Hum. Genet..

[84] Xie G., Liang C., Yu H., Zhang Q. (2021). Association between polymorphisms of collagen genes and susceptibility to intervertebral disc degeneration: A meta-analysis.. J. Orthop. Surg. Res..

[85] Knoeringer M., Reinke A., Trappe A.E., Schlegel J. (2008). Absence of the mutated Trp2 allele but a common polymorphism of the COL9A2 collagen gene is associated with early recurrence after lumbar discectomy in a German population.. Eur. Spine J..

[86] Meng T., Ren Q., Wang J.M., Shi H., Zhang S.T., Liu M.T. (2016). Association between COL9A2 Gln326Arg mutations and the development of intervertebral disc disease in a Chinese population.. Genet. Mol. Res..

[87] Paassilta P., Lohiniva J., Göring H.H., Perälä M., Räinä S.S., Karppinen J., Hakala M., Palm T., Kröger H., Kaitila I., Vanharanta H., Ott J., Ala-Kokko L. (2001). Identification of a novel common genetic risk factor for lumbar disk disease.. JAMA.

[88] Chen T., Li G-B., Liang K-Y., Jia S-Q. (2013). Correlation between lumbar disc degeneration and COL9A2 gene single nucleotide polymorphisms. Chin J.. Tissue Eng. Res..

[89] Wu H., Wang S., Chen W., Zhan X., Xiao Z., Jiang H., Wei Q., Collagen I.X. (2018). Collagen IX gene polymorphisms and lumbar disc degeneration: A systematic review and meta-analysis.. J. Orthop. Surg. Res..

[90] Markova T., Sparber P., Borovikov A., Nagornova T., Dadali E. (2021). Clinical and genetic characterization of autosomal recessive stickler syndrome caused by novel compound heterozygous mutations in the COL9A3 gene.. Mol. Genet. Genomic Med..

[91] Paassilta P., Pihlajamaa T., Annunen S., Brewton R.G., Wood B.M., Johnson C.C., Liu J., Gong Y., Warman M.L., Prockop D.J., Mayne R., Ala-Kokko L. (1999). Complete sequence of the 23-kilobase human COL9A3 gene. Detection of Gly-X-Y triplet deletions that represent neutral variants.. J. Biol. Chem..

[92] Romero-Ortuno R., Kenny R.A., McManus R. (2020). Collagens and elastin genetic variations and their potential role in aging-related diseases and longevity in humans.. Exp. Gerontol..

[93] Biczo A., Bereczki F., Koch K., Varga P.P., Urban J., Fairbank J., Heywood C., Sivan S., Roberts S., Neidlinger-Wilke C., Kaprio J., Battie M.C., Kletsas D., Ito K., Huyghe J., Brayda-Bruno M., Velikonja N.K., Lazary A. (2022). Genetic variants of interleukin 1B and 6 are associated with clinical outcome of surgically treated lumbar degenerative disc disease.. BMC Musculoskelet. Disord..

[94] Zhang P., Chang B.G. (2020). Inhibitory effect on the apoptosis of disc nucleus pulposus cells in rats by silencing COL9A3 gene to mediate MAPK signaling pathway: A study on the function and mechanism.. Eur. Rev. Med. Pharmacol. Sci..

[95] Solovieva S., Lohiniva J., Leino-Arjas P., Raininko R., Luoma K., Ala-Kokko L., Riihimäki H. (2002). COL9A3 gene polymorphism and obesity in intervertebral disc degeneration of the lumbar spine: Evidence of gene-environment interaction.. Spine.

[96] Solovieva S., Lohiniva J., Leino-Arjas P., Raininko R., Luoma K., Ala-Kokko L., Riihimäki H. (2006). Intervertebral disc degeneration in relation to the COL9A3 and the IL-1ß gene polymorphisms.. Eur. Spine J..

[97] Eskola P.J., Kjaer P., Daavittila I.M., Solovieva S., Okuloff A., Sorensen J.S., Wedderkopp N., Ala-Kokko L., Männikkö M., Karppinen J.I. (2010). Genetic risk factors of disc degeneration among 12-14-year-old Danish children: A population study.. Int. J. Mol. Epidemiol. Genet..

[98] Yang X., Jia H., Xing W., Li F., Li M., Sun K., Zhu Y. (2020). Multiple variants in collagen genes are associated with the susceptibility to lumbar disc herniation in the Chinese population.. Eur. Spine J..

[99] Jiang L., Wang C., Ye Z., Hu Q. (2024). A novel missense COL9A3 variant in a pedigree with multiple lumbar disc herniation.. J. Orthop. Surg. Res..

[100] Luo Y.Y., Karsdal M.A., Karsdal M.A. (2016). Type XI Collagen.. Biochemistry of Collagens, Laminins and Elastin..

[101] Nallanthighal S., Heiserman J.P., Cheon D.J. (2021). Collagen Type XI Alpha 1 (COL11A1): A Novel Biomarker and a Key Player in Cancer.. Cancers.

[102] Kuivaniemi H., Tromp G., Prockop D.J. (1997). Mutations in fibrillar collagens (types I, II, III, and XI), fibril-associated collagen (type IX), and network-forming collagen (type X) cause a spectrum of diseases of bone, cartilage, and blood vessels.. Hum. Mutat..

[103] Mio F., Chiba K., Hirose Y., Kawaguchi Y., Mikami Y., Oya T., Mori M., Kamata M., Matsumoto M., Ozaki K., Tanaka T., Takahashi A., Kubo T., Kimura T., Toyama Y., Ikegawa S. (2007). A functional polymorphism in COL11A1, which encodes the alpha 1 chain of type XI collagen, is associated with susceptibility to lumbar disc herniation.. Am. J. Hum. Genet..

[104] Vijzelaar R., Waller S., Errami A., Donaldson A., Lourenco T., Rodrigues M., McConnell V., Fincham G., Snead M., Richards A. (2013). Deletions within COL11A1in Type 2 stickler syndrome detected by multiplex ligation-dependent probe amplification (MLPA).. BMC Med. Genet..

[105] Fernandes R.J., Weis M., Scott M.A., Seegmiller R.E., Eyre D.R. (2007). Collagen XI chain misassembly in cartilage of the chondrodysplasia (Cho) mouse.. Matrix Biol. J. Int. Soc. Matrix Biol..

[106] Rodriguez R.R., Seegmiller R.E., Stark M.R., Bridgewater L.C. (2004). A type XI collagen mutation leads to increased degradation of type II collagen in articular cartilage.. Osteoarthritis Cartilage.

[107] Sun M., Luo E.Y., Adams S.M., Adams T., Ye Y., Shetye S.S., Soslowsky L.J., Birk D.E., Collagen X.I. (2020). Collagen XI regulates the acquisition of collagen fibril structure, organization and functional properties in tendon.. Matrix Biol..

[108] Li Y., Lacerda D.A., Warman M.L., Beier D.R., Yoshioka H., Ninomiya Y., Oxford J.T., Morris N.P., Andrikopoulos K., Lifferth G.D. (1995). A fibrillar collagen gene, Col11a1, is essential for skeletal morphogenesis.. Cell.

[109] Raine E.V.A., Dodd A.W., Reynard L.N., Loughlin J. (2013). Allelic expression analysis of the osteoarthritis susceptibility gene COL11A1 in human joint tissues.. BMC Musculoskelet. Disord..

[110] Liu W., Sun G., Guo L., Wang L., Fan W., Lang M., Chen D., Yi X. (2017). A genetic variant in COL11A1 is functionally associated with lumbar disc herniation in Chinese population.. J. Genet..

[111] Koyama K., Nakazato K., Maeda S., Kikuchi N., Matsumoto S., Hiranuma K. (2018). Association of COL11A1 4603C/T polymorphism with cervical disc degeneration in collegiate wrestlers.. J. Sports Med. Phys. Fitness.

[112] Sambrook P.N., MacGregor A.J., Spector T.D. (1999). Genetic influences on cervical and lumbar disc degeneration: A magnetic resonance imaging study in twins.. Arthritis Rheum..

[113] Koyama K., Nakazato K., Min S., Gushiken K., Hatakeda Y., Seo K., Hiranuma K. (2012). COL11A1 gene is associated with limbus vertebra in gymnasts.. Int. J. Sports Med..

[114] Wu F., Huang X., Zhang Z., Shao Z. (2020). A meta-analysis assessing the association between COL11A1 and GDF5 genetic variants and intervertebral disc degeneration susceptibility.. Spine.

[115] (2024). Reference SNP (rs) Report.. https://www.ncbi.nlm.nih.gov/snp/rs1337185.

[116] Jiang H., Yang Q., Jiang J., Zhan X., Xiao Z. (2017). Association between COL11A1 (rs1337185) and ADAMTS5 (rs162509) gene polymorphisms and lumbar spine pathologies in Chinese Han population: An observational study.. BMJ Open.

[117] Rajasekaran S., Kanna R.M., Senthil N., Raveendran M., Cheung K.M.C., Chan D., Subramaniam S., Shetty A.P. (2013). Phenotype variations affect genetic association studies of degenerative disc disease: Conclusions of analysis of genetic association of 58 single nucleotide polymorphisms with highly specific phenotypes for disc degeneration in 332 subjects.. Spine J..

[118] COL11A2 - collagen type XI alpha 2 chain. (2024). https://www.ncbi.nlm.nih.gov/datasets/gene/1302/.

[119] Bao P., Jiang L., Liu G., Li Y., Chen X., Wang M. (2025). Two cases of autosomal dominant familial short stature associated with COL11A2 gene variant and the therapeutic response to recombinant human growth hormone.. Transl. Pediatr..

[120] Rebello D., Wohler E., Erfani V., Li G., Aguilera A.N., Santiago-Cornier A., Zhao S., Hwang S.W., Steiner R.D., Zhang T.J., Gurnett C.A., Raggio C., Wu N., Sobreira N., Giampietro P.F., Ciruna B. (2023). COL11A2 as a candidate gene for vertebral malformations and congenital scoliosis.. Hum. Mol. Genet..

[121] Lawrence E.A., Kague E., Aggleton J.A., Harniman R.L., Roddy K.A., Hammond C.L. (2018). The mechanical impact of col11a2 loss on joints; col11a2 mutant zebrafish show changes to joint development and function, which leads to early-onset osteoarthritis.. Philos. Trans R Soc. Lond B Biol. Sci..

[122] Urabe K., Jingushi S., Ikenoue T., Okazaki K., Sakai H., Li C., Iwamoto Y. (2001). Immature osteoblastic cells express the pro‐α2(XI) collagen gene during bone formation in vitro and in vivo.. J. Orthop. Res..

[123] Acke F.R., Malfait F., Vanakker O.M., Steyaert W., De Leeneer K., Mortier G., Dhooge I., De Paepe A., De Leenheer E.M.R., Coucke P.J. (2014). Novel pathogenic COL11A1/COL11A2 variants in Stickler syndrome detected by targeted NGS and exome sequencing.. Mol. Genet. Metab..

[124] Sheu J.J., Lin Y.J., Chang J.S., Wan L., Chen S.Y., Huang Y.C., Chan C., Chiu I.W., Tsai F.J. (2010). Association of COL11A2 polymorphism with susceptibility to Kawasaki disease and development of coronary artery lesions.. Int. J. Immunogenet..

[125] Tompson S.W., Bacino C.A., Safina N.P., Bober M.B., Proud V.K., Funari T., Wangler M.F., Nevarez L., Ala-Kokko L., Wilcox W.R., Eyre D.R., Krakow D., Cohn D.H. (2010). Fibrochondrogenesis results from mutations in the COL11A1 type XI collagen gene.. Am. J. Hum. Genet..

[126] Yang X., Jia H., Xing W., Li F., Li M., Sun K., Zhu Y. (2019). Genetic variants in COL11A2 of lumbar disk degeneration among Chinese Han population.. Mol. Genet. Genomic Med..

[127] Xu R., Jiang X., Lu J., Wang K., Sun Y., Zhang Y. (2020). Genetic variant of COL11A2 gene is functionally associated with developmental dysplasia of the hip in Chinese Han population.. Aging (Albany NY).

[128] Maeda S., Ishidou Y., Koga H., Taketomi E., Ikari K., Komiya S., Takeda J., Sakou T., Inoue I. (2001). Functional impact of human collagen alpha2(XI) gene polymorphism in pathogenesis of ossification of the posterior longitudinal ligament of the spine.. J. Bone Miner. Res..

[129] Noponen-Hietala N., Kyllönen E., Männikkö M., Ilkko E., Karppinen J., Ott J., Ala-Kokko L. (2003). Sequence variations in the collagen IX and XI genes are associated with degenerative lumbar spinal stenosis.. Ann. Rheum. Dis..

[130] Virtanen I.M., Karppinen J., Taimela S., Ott J., Barral S., Kaikkonen K., Heikkilä O., Mutanen P., Noponen N., Männikkö M., Tervonen O., Natri A., Ala-Kokko L. (2007). Occupational and genetic risk factors associated with intervertebral disc disease.. Spine.

[131] Williams F.M.K., Bansal A.T., van Meurs J.B., Bell J.T., Meulenbelt I., Suri P., Rivadeneira F., Sambrook P.N., Hofman A., Bierma-Zeinstra S., Menni C., Kloppenburg M., Slagboom P.E., Hunter D.J., MacGregor A.J., Uitterlinden A.G., Spector T.D. (2013). Novel genetic variants associated with lumbar disc degeneration in northern Europeans: A meta-analysis of 4600 subjects.. Ann. Rheum. Dis..

[132] Lin C.Y., Crowley S.T., Uchida S., Komaki Y., Kataoka K., Itaka K. (2019). Treatment of Intervertebral disk disease by the administration of mRNA encoding a cartilage-anabolic transcription factor.. Mol. Ther. Nucleic Acids.

[133] Chang C.C., Tsou H.K., Chang H.H., Chan L.Y., Zhuo G.Y., Maeda T., Lin C.Y. (2022). Runx1 messenger RNA delivered by polyplex nanomicelles alleviate spinal disc hydration loss in a rat disc degeneration model.. Int. J. Mol. Sci..

[134] Krupkova O., Cambria E., Besse L., Besse A., Bowles R., Wuertz-Kozak K. (2018). The potential of CRISPR/Cas9 genome editing for the study and treatment of intervertebral disc pathologies.. JOR Spine.

[135] Seidl C.I., Fulga T.A., Murphy C.L. (2019). CRISPR-Cas9 targeting of MMP13 in human chondrocytes leads to significantly reduced levels of the metalloproteinase and enhanced type II collagen accumulation.. Osteoarthritis Cartilage.

[136] Takeoka Y., Yurube T., Nishida K. (2020). Gene therapy approach for intervertebral disc degeneration: An update.. Neurospine.

[137] Levis H., Lewis C., Fainor M., Lawal A., Stockham E., Weston J., Farhang N., Gullbrand S.E., Bowles R.D. (2025). Targeted CRISPR regulation of ZNF865 enhances stem cell cartilage deposition, tissue maturation rates, and mechanical properties in engineered intervertebral discs.. Acta Biomater..

[138] Daliri K., Hescheler J., Newby G.A., Clement K., Liu D.R., Pfannkuche K. (2025). Modulating collagen I expression in fibroblasts by CRISPR-Cas9 base editing of the collagen 1A1 promoter.. Int. J. Mol. Sci..

[139] Li W., Li X.H., Gao Y., Xiong C.J., Tang Z.Z. (2023). Emerging roles of RNA binding proteins in intervertebral disc degeneration and osteoarthritis.. Orthop. Surg..

[140] Lin Z., Lu F., Ma X., Xia X., Zou F., Jiang J. (2021). Roles of circular RNAs in the pathogenesis of intervertebral disc degeneration (Review).. Exp. Ther. Med..

[141] Li X., Chang H., Cai F., Zhang Y., Li A., Yang X., Cai Z., Cui W., Liu X. (2024). Progress of gene‐functionalized regenerative material repair intervertebral disc degeneration.. Small Sci..

[142] Jiménez Calvente C., Sehgal A., Popov Y., Kim Y.O., Zevallos V., Sahin U., Diken M., Schuppan D. (2015). Specific hepatic delivery of procollagen α1(I) small interfering RNA in lipid‐like nanoparticles resolves liver fibrosis.. Hepatology.

[143] Wang S., Wei X., Sun X., Chen C., Zhou J., Zhang G., Wu H., Guo B., Wei L. (2018). A novel therapeutic strategy for cartilage diseases based on lipid nanoparticle-RNAi delivery system.. Int. J. Nanomedicine.

[144] Wijesinghe S.N., Lindsay M.A., Jones S.W. (2021). Oligonucleotide therapies in the treatment of arthritis: A narrative review.. Biomedicines.

[145] Vasiliadis E.S., Pneumaticos S.G., Evangelopoulos D.S., Papavassiliou A.G. (2014). Biologic treatment of mild and moderate intervertebral disc degeneration.. Mol. Med..

